# Evaluation of Aging Effects on Asphalt Binders and Pavements: Rheological Responses to Rejuvenators and Numerical Analysis of Polymer Modification

**DOI:** 10.3390/polym18060759

**Published:** 2026-03-20

**Authors:** Ahmet Sertac Karakas

**Affiliations:** Construction and Technical Department, Istanbul University, Istanbul 34116, Turkey; skarakas@istanbul.edu.tr

**Keywords:** binder, rejuvenator, aging, finite element model, Plaxis

## Abstract

The restricted availability of raw materials underscores the significance of recycling asphalt materials that have reached the end of their service life, facilitating their reuse with additives for economic and sustainability benefits. The study includes both empirical investigations and numerical analyses. Empirical studies were conducted in four stages to evaluate the binder and mixture. First, the rheological properties of binders obtained from various sources were assessed in both unmodified and modified states. Second, the binders were subjected to different levels of aging. Third, the presence of additives in the binders was investigated. In the final stage, the analysis of asphalt pavement layers was conducted using the finite element method (FEM) for both modified and unmodified binders. Performance tests were carried out to evaluate the binder’s properties, and physical examinations were conducted to compare these properties. The binders were tested under both unaged and aged conditions using linear amplitude sweep (LAS), frequency sweep (FS), multiple stress creep recovery (MSCR), and bending beam rheometer (BBR) tests. The results indicated that aging increased the stiffness of the binders, regardless of their source. Additionally, the introduction of a rejuvenator reduced the binder’s stiffness, particularly at low temperatures. Findings showed that the growth rate (GR) and rutting parameters increased with binder aging, while the frequency decreased. The R^2^ value of 0.92 demonstrates a strong correlation between the parameters. Polymer-modified binders demonstrated superior deformation resistance and higher stiffness stability. Overall, aging reduced asphalt flexibility, whereas modified binders improved long-term pavement deformation performance.

## 1. Introduction

Asphalts are exposed to environmental conditions and aging from the construction stage through application. During its service life, aging and fatigue effects are observed due to traffic and environmental conditions, such as climate. Oxidation of the binder’s chemical structure is the most common form of degradation in this respect. With aging, significant problems might occur in the asphalt coating. The physical properties and chemical structure of asphalt change due to traffic effects and environmental conditions, including temperature changes, humidity, ultraviolet irradiation, and heat radiation. In asphalt pavements, problems such as moisture damage, hardening, stripping, fatigue cracking, potholes, and raveling occur after aging due to external effects. For this reason, additives are used to improve the engineering properties of bitumen and mitigate hardness, peeling, low- and high-temperature cracking, accelerated fatigue cracking, and humidity sensitivity. Additive materials are mixed with bituminous and hot bituminous mixtures. The most commonly used modifier is a polymer. Rejuvenators are used to make asphalt resistant to fatigue and aging, therefore increasing its service life. Various rejuvenators are used with asphalt, such as Hydroline H90T, Hydrogreen^®^S, and Kendex.

Bitumen, the raw material for asphalt, requires careful assessment given increased demand and declining reserves. Although the raw materials processed in refineries have the same penetration rate, the asphaltene content varies with the primary source and location, resulting in different properties. Asphalt, which constitutes the main component of bitumen, is found in varying proportions in the Middle East, Africa, and America, where bitumen is supplied as a raw material. In many refineries across five regions in the USA, asphalt raw material is processed to produce bitumen. The raw materials from these five regions are supplied by North and South America, Africa, and the Middle East. Therefore, the raw materials processed in refineries differ, reflecting the properties of the regions from which they are procured.

Bitumen materials obtained from different sources, possessing similar engineering properties and the same penetration degree, exhibit different physical and chemical engineering characteristics due to the aging processes and the modifier additives used for improvement. Furthermore, there are gaps in the literature regarding the evaluation of the field performance of asphalt pavements under different climatic and traffic conditions, and the support of empirical experimental data with numerical analyses for future predictions. The findings of this study provide support in addressing these shortcomings in the literature.

The study was conducted to understand asphalt’s behavior under environmental factors. In the study, original and modified PG64-22 and PG70-22 binders were used to investigate the performance properties of asphalt against aging. The original binder aging was simulated in the laboratory, and its post-rejuvenation properties and aging behavior were analyzed using modifiers. Furthermore, by creating a scenario, the behavior of asphalt pavement on different soil layers under traffic load was examined, and the mechanical properties of asphalt pavement were investigated under traffic and other environmental conditions over 1 year for both modified and unmodified conditions. The study has added innovation in both the effects of various types of rejuvenators on the performance properties of asphalt and the determination of stress–strain relations of polymer-modified asphalt pavement under environmental and traffic conditions.

Despite the extensive body of literature on asphalt aging, polymer modification, and rejuvenation, important gaps remain. In particular, the aging sensitivity and rejuvenator effectiveness of binders with similar performance grades but different crude oil origins have not been systematically compared. Moreover, the majority of existing studies rely solely on laboratory experiments, while the integration of experimental findings with numerical pavement modeling under realistic traffic and environmental conditions is still limited. Therefore, the objective of this study is to evaluate the aging and rejuvenation behavior of original and modified PG64-22 and PG70-22 binders through laboratory aging simulations, and to investigate the corresponding mechanical response of asphalt pavements using numerical analysis. By combining rheological characterization with stress–strain evaluation of pavement structures under traffic loading and environmental effects, this study aims to provide a more comprehensive understanding of the long-term performance of polymer-modified and rejuvenated asphalt pavements.

## 2. Literature

Various mechanisms occur in the in-service state of asphalt pavements, which damage their viscoelastic properties. Aging is well accepted in the literature as one of the most complex mechanisms, if not the most complicated, that impinges visco-elasticity and other vital engineering properties of asphalt pavement and that ultimately induces critical pavement distresses such as stiffening, embrittlement, and lower fatigue resistance [1,2,3,4,5,6].

Asphalt binder is the only viscoelastic constituent in asphalt pavement, and it hardens over time. The main reason for this hardening is changes in the chemical composition and molecular structure of the asphalt binder due to its inevitable reactions with various environmental factors, such as oxygen, ultraviolet irradiation, and thermal diffusion. Unfortunately, this action often occurs in a conjoint environment, making it even more challenging for researchers to understand aging better. In addition, asphalt mix design parameters such as pavement air void content, aggregate petrography, porosity, and morphology can accelerate or delay age hardening, as these factors affect the rate at which oxygen can diffuse into the pavement layers. The more of these factors are present in the pavement or asphalt-aggregate mixture system, the greater the oxygen and thermal diffusion, which consequently alter the chemical integrity of the binder [7].

Abbas et al. [8] investigated the effects of recycling asphalt shingles (RAS) on the physical and chemical properties of asphalt binders. PG 58-28 binder was used at different percentages (0%, 5%, 7%, and 10%) with RAS binder. The chemical properties of the binders were evaluated using gel permeation chromatography (GPC) and Fourier transform infrared spectroscopy (FTIR). Performance tests were conducted to determine the binder’s low-, medium-, and high-temperature properties. The test results showed a higher susceptibility to early thermal cracking but also showed resistance to permanent deformation with the addition of the RAS. It was observed that increasing the RAS ratio accelerated asphalt aging. Further aging was observed with the addition of RAS recycling material to the binders and increasing the ratio [8].

Abdullah et al. studied nanotechnology and evaluated the structural and morphological characteristics of modifier binders containing nanoparticles. Various modifiers are used to improve asphalt’s properties. Modified binders incorporating nanoparticles are favored due to their influence on the engineering characteristics of asphalt. Polymeric nanocomposites are considered nanotechnology materials for improved pavement performance. In this study, asphalt binder and mixture were studied to understand nanoparticle types, dosages, modified procedures, and nanomaterial properties [9].

Abdullah et al. [10] investigated the rheological effects of aging asphalt binder modified with montmorillonite (MMT). The study found that using the MMT modifier improved fatigue resistance, viscosity, and rutting resistance. In the FTIR test, the additional MMT appeared to delay the aging properties.

Aguirre et al. [11] assessed the micro-encapsulation of asphalt rejuvenators using lamine-formaldehyde. Three binder types were used in the experimental study. PG70-22 and PG76-22 virgin asphalt binders were aged in the laboratory, and reclaimed asphalt pavement (RAP) was extracted for use in the experimental research. The rejuvenator with PG70-22 was effective at both high and low temperatures, and the high-temperature grade of the RAP binder and the low-temperature grade of the PG76-22 binder were restored.

Ahmedzade et al. [12] examined waste polymer for use in a highway application. Recycled polypropylene was determined to be a waste polymer for modified bitumen. Five different ratios of modified bitumen were investigated for their chemical and physical properties. FTIR spectroscopy, fluorescence microscopy, conventional tests, short- and long-term aging procedures, rotational viscosity, dynamic shear, and bending beam rheometer tests were performed to study the modifier binder. As a test result, the use of modified bitumen was found to be convenient for enhancing asphalt properties.

Airey et al. [7] investigated the rheological properties of asphalt binder to assess not only temperature but also the time regime in terms of the material’s dynamic (oscillatory) viscoelastic response. According to the test results, polymer- and rubber-modified binders improved the viscoelastic properties, with increased complex shear modules observed exclusively at high temperatures and low frequencies. The rheological properties of bitumen modified with polymer and waste rubber were improved compared to traditional bitumen. The synthetic binders differed significantly from both conventional bituminous binders and polymer- and crumb-rubber-modified binders.

Al-Azri et al. [13] examined binder oxidation in pavements. Texas highway pavements were selected for extensive investigations. Field aging was compared with laboratory aging of the neat binder. The level of hardening of pavement was observed both near the surface and 4 to 6 inches below the surface.

Alavi et al. investigated the effects of oxidative aging of asphalt mixtures using carbonyl groups and proposed a new approach. A good correlation was established between the parameters of the asphalt mixture and the carbonyl and the continuous relaxation spectrum, and a positive effect of the carbonyl was observed [14].

Ali et al. investigated the effects of aging on the low- and high-temperature performance of asphalt using asphalt binders modified with five different rejuvenators. Naphthenic oil, paraffinic oil, aromatic extracts, tall oil, and oleic acid have been used as rejuvenators in asphalt binders. Using different rejuvenation methods, the short-term aging performance (2–6 h) was investigated under low- and high-temperature conditions. All rejuvenated binders had lower performance grades than those without rejuvenated binders. The use of RAP up to 45% did not affect aging [15].

Al-Rub et al. [16] proposed an aging model based on a mechanistic-phenomenological approach to oxidative aging, or oxidative hardening, to capture the elastic, viscoplastic, and visco-damage responses of aging-susceptible bituminous materials. The proposed aging model was successfully applied to predict the effects of aging on the mechanical behavior of various materials, including polymers, soft materials, biomaterials, and asphalt binders.

Apeagyei examined the rheological properties of asphalt binders undergoing oxidative aging. The use of antioxidants in reducing the effects of aging in asphalt was analyzed. It was determined that some additives, such as vitamin E, Irganox 1010, Irgafos P-EPQ, carbon black, hydrated lime, and DLTDP/furfural, which are used as antioxidants in asphalt, improve performance [17].

Araujo et al. [18] examined the effects of modifiers, such as SBS, RET, and PPA, on asphalt aging. SBS-, PPA-, and RET-modified asphalt indicated higher segregation resistance and greater thermal stability during aging.

Arega et al. [19] investigated the effects of using wax in asphalt binder as a modifier and observed a reduction in hardness and increased susceptibility to permanent deformation.

Asli et al. [20] used waste edible oil (WCO) as a modifier to improve bitumen properties. There was no difference between the softening point and viscosity tests before and after modification.

Azzam et al. [21] compared oil shale fill aggregate and bituminous hot mix (HMA) containing limestone aggregate. It was observed that the petroleum shale aggregate additive was less effective at resisting rutting in the short term than the limestone aggregate mixture. Still, it provides significant long-term contributions to fatigue resistance.

Baek et al. [22] studied the effects of asphalt mixture on oxidative aging and fatigue resistance. Aging was found to negatively affect the performance of asphalt concrete pavements prepared with hot bituminous mixtures.

Bahia et al. [23] determined the nonlinear viscoelastic and fatigue properties of asphalt binders. It was observed that factors such as additive type and binder composition used during aging and fatigue processes are effective.

Bahia et al. [24] used new tests to evaluate stability, the nature of the modifier, and short-term aging of modified binders. The results indicate that the modification has a significant effect on aging, as shown by time comparisons of aging for several modified asphalts with and without RTFOT modification.

Bennert et al. [25] used refined engine oil base (REOB) to evaluate the fatigue performance of asphalts. It was observed that the hardening and aging processes accelerated with increasing REOB additive content in the binder.

Bhasin et al. [26] investigated the effects of temperature and aging on the rate of intrinsic healing of asphalt binders. The aging performance of several asphalt kinds at different temperatures was examined. It was demonstrated that the properties of the asphalt binder change with increasing temperature and aging.

Binbin et al. [27] examined the influence of waste edible vegetable oil on the high-temperature characteristics of asphalts of varying ages. The results indicated that waste edible vegetable oil slightly reduced the viscosity value of aged asphalt. The complex modulus of binders decreased, reducing the rutting resistance factor of the rejuvenated asphalt due to the addition of waste edible vegetable oil.

Bommeria et al. [28] determined the effect of saturated aging on the fatigue behavior of asphalt pavements. In the study, fatigue tests were applied before and after aging. The test results showed that the reduction in the mix’s strength due to moisture aging is significant only for up to 25 h of aging. It was determined that the indirect tensile strength of the mixture and the modulus of elasticity of AC11 and SMA mixtures decreased after aging.

Bressi et al. [29] examined the impacts of different aging levels on binder rheology. Their tests were conducted at different aging levels and temperatures. The stiffness changed significantly across aging levels, and its variance became more sensitive to temperature changes.

Hill et al. [30] studied the low-temperature properties of RAP and virgin bio-modified asphalt binder. It was observed that BMB mixtures had higher fracture energies than HMA at all RAP levels. It was understood that BMB RAP mixtures exhibited low-temperature cracking behavior superior to that of HMA mixtures.

Canestrari et al. [31] evaluated the fatigue and recovery performance of aged binders with the SBS modifier and found that it improved performance. As regards test results, the SBS polymer enhanced the self-healing capacity. Long-term aging improved both fatigue resistance and self-healing ability, without adversely affecting virgin binder performance.

Chen et al. [32] studied the engineering properties of RAP and the use of aged bitumen. A negative deviation was observed with the use of a rejuvenating agent; viscosity at the standard mixing temperature and the time-temperature principle were found to be sufficient to characterize the aged bitumen behavior.

Colbert and You [33] examined the effects of recycled asphalt on the short- and long-term aging of a PG58-28 binder, using different RAP binder ratios. Rotational viscosity and frequency sweep tests were conducted with a Brookfield viscometer and a dynamic shear rheometer, respectively. According to the test results, increasing the RAP ratio and applying additional aging conditions increased the stiffness and viscosity of RAP binder blends.

Hamit et al. [34] used SBS polymers as additives. It was determined that the use of ground SBS in hot bituminous mixtures gives better results [34].

Rejuvenators are used to improve binder properties in recycled asphalts. Pongamia oil and compound castor oil rejuvenators were used to increase the rate of RAP utilization, and positive effects on asphalt performance were observed [35]. The use of modifiers in HMAs has positive effects on performance against deterioration. SBS block copolymers are one of the most widely used modifiers to improve binder performance. Studies indicate that SBS polymers exhibit significant resistance to rutting, fatigue, and cracking under different environmental conditions [36,37,38,39,40,41].

Qadi et al. [42] used liquid anti strip (LA-Stripe), polyphosphoric acid (PPA), and slaked lime as modifiers in their studies. The use of a liquid anti-stripping agent and hydrated lime had an adverse effect by reducing the moisture sensitivity of asphalt mixtures [42].

Yao et al. [43] demonstrated that, using an FTIR-based blending model, RAP content, gradation type, asphalt content, mixing duration, mixing temperature, RAP preheating temperature, and mixing sequence positively influence the blending degree of recycled asphalt mixtures.

In studies, styrene–butadiene–styrene copolymers (SBSC) have been used as modifiers due to their significant contribution to the mechanical properties and performance of asphalt [44,45,46]. Deformations of asphalt pavements can occur under environmental conditions and repeated loads [47]. The finite element method is used to determine the rutting depth on the road surface caused by repeated loading over time in asphalt layers, and numerical analyses are performed using finite element programs [48,49].

Most previous studies have primarily investigated the effects of aging, polymer modification, or rejuvenation on asphalt binders in isolation. However, a comprehensive evaluation that simultaneously considers different PG64-22 binder sources, rejuvenator dosage, and the pavement response of SBS-modified binders under combined environmental and traffic loading remains limited. Although the use of rejuvenators and SBS polymers is widely recognized as an effective approach to extending the service life of asphalt pavements, existing studies indicate that binders possessing identical engineering grades but sourced from different crude origins may exhibit significantly different performance characteristics. Furthermore, research addressing the stress and deformation behavior of SBS-modified asphalt pavements constructed over different subgrade conditions and subjected to time-dependent traffic and climatic effects through numerical analysis is still scarce.

To address these gaps, this study comparatively evaluates the performance of aged and rejuvenated asphalt binders with identical and varying penetration grades obtained from different sources, with particular emphasis on their rheological and macroscopic chemical characteristics. In addition, the structural response of SBS-modified asphalt pavements under traffic and climatic conditions is investigated using finite element–based numerical analyses, enabling a detailed assessment of stress–deformation behavior over different service time periods.

## 3. Aging Behavior of Asphalt Using Rejuvenator and SBS-Modified Asphalt

### 3.1. Behavior of Asphalt and Factors That Affect Asphalt Aging

Recent advances in the literature have increasingly emphasized the coupled effects of rejuvenators and SBS modification on the aging behavior of asphalt binders. Previous studies indicate that rejuvenators can partially mitigate aging-induced compositional changes by replenishing depleted light fractions and enhancing molecular mobility, thereby enabling a limited recovery of rheological properties. However, both the magnitude and durability of this recovery are strongly dependent on the rejuvenator type, dosage, and the prior aging history of the binder [50,51,52,53,54]. In contrast, SBS modification has been reported to influence the aging process primarily by altering oxidation kinetics and moderating stiffness evolution, contributing to improved resistance to aging-related hardening and enhanced retention of elastic response under long-term environmental and traffic loading. Nevertheless, the effectiveness of SBS modification remains sensitive to polymer content, dispersion quality, and binder source [55,56].

**Figure 1:**
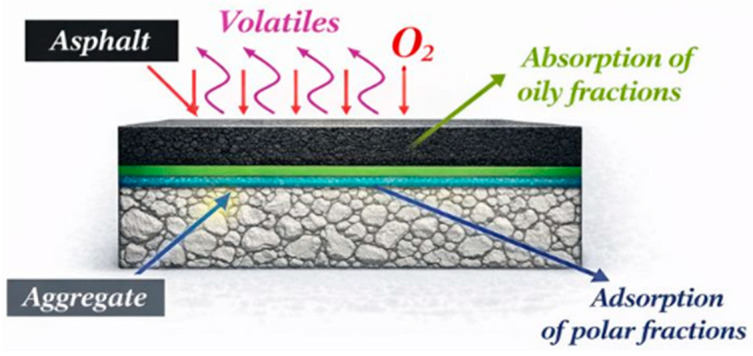
Illustration of the asphalt aging process because of volatilization, oxidation, absorption, and adsorption, reproduced from [59], Strategic Highway Research Program (SHRP), 1989.

Asphalt aging is often characterized by deterioration of adhesive qualities, a gradual increase in viscosity, and major changes in mechanical modulus. Wu and Khalid [57,58] integrated foundational studies and established that asphalt aging is predominantly determined by the binder’s compositional change and microstructural reorganization. These aging mechanisms include steric hardening, oxidation-induced molecular rearrangement due to interactions with ambient oxygen, and the slow depletion of low-molecular-weight elements via volatilization or adsorption onto mineral aggregate surfaces. Table 1 shows the aging-related parameters compiled by Wu and Khalid, whereas Figure 1 depicts the microstructural evolution of asphalt during the aging process. Asphalt cement has a fluid, elastic, and solid structure. The molecular structure of asphalt changes with its oxidation. The performance properties of asphalt binder change with temperature, repeated loading, and aging. In asphalt pavements, aging, hardening, and viscosity increase are observed during the service life under environmental conditions.

### 3.2. Bitumen Modification

Bitumen plays an essential role in resisting cracking and permanent deformation in pavement layers due to its viscoelastic rheological structure. The amount of deformation in asphalt pavement varies with traffic load and environmental conditions, such as temperature changes. Common types of deterioration in asphalt pavements include moisture damage, thermal cracking, and fatigue cracking. Polymers are widely used to increase the strength and service life of asphalt pavements.

Distress in asphalt pavement can be caused by traffic and environmental conditions, and thanks to the modifiers used, it can reduce deformation, moisture damage, creep, fatigue, and noise [60,61].

Modified bitumen prepared with SBS additives is widely used to improve the physical and mechanical properties of HMAs. When the SBS structure is examined, it maintains its properties across different temperatures (from −40 °C to +80 °C). Because it has a thermoplastic-elastomer structure, it is not affected by heating or cooling. It creates an elastic, flexible structure with a modifier and a strong network structure when it becomes fluid.

The types of bitumen modification are given in Table 2.

Additives used in the modification of bituminous and bituminous mixtures and the benefits according to the shape of deterioration are given in Table 3.

### 3.3. Application of Rejuvenators in Asphalt

Oxidative aging in asphalt pavements induces a progressive increase in binder stiffness over the service life, which may lead to embrittlement and susceptibility to cracking under traffic loading. The depletion of virgin aggregates and bitumen has necessitated the integration of recycled materials, such as reclaimed asphalt pavement (RAP) and recycled asphalt shingles (RAS), into asphalt mixtures. These recycled constituents significantly influence the rheological properties of the binder, altering viscoelastic behavior, fatigue resistance, and temperature susceptibility, thereby requiring careful adjustment of mixture design parameters to ensure performance equivalence with conventional mixtures.

Rejuvenating agents have emerged as a critical strategy to counteract the deleterious effects of aging. These additives restore maltene fractions and reduce asphaltene dominance in aged binders, effectively lowering stiffness and enhancing ductility. By mitigating oxidative hardening, rejuvenators improve resistance to thermal and fatigue cracking, slow the progression of embrittlement, and enhance durability under environmental loading, including ultraviolet exposure and moisture-induced damage. The application of rejuvenated binders contributes to extended pavement service life, reduced maintenance interventions, and economic efficiency, while maintaining or improving key rheological characteristics such as complex modulus, phase angle, and susceptibility to permanent deformation.

### 3.4. Rejuvenating Agents

Rejuvenating agents play a critical role in enhancing the performance characteristics of conventional asphalt binders, especially when integrated with recycled materials. Their application as additives contributes to significant improvements in both the mechanical and physical behavior of asphalt mixtures:

Improved Workability and Compaction: The addition of rejuvenators enhances the flow properties of hot mix asphalt (HMA) containing recycled asphalt materials, allowing for more uniform compaction and consistent density during pavement construction. This ensures a more homogenous mixture and minimizes void-related defects.

Enhanced Low-Temperature Flexibility: Rejuvenators increase the ductility and fracture resistance of binders at low temperatures, reducing the likelihood of thermal cracking. This effect is particularly important in climates with extreme cold or for mixtures with high recycled content.

Increased High-Temperature Stability: By moderating stiffness gains in asphalt binders, rejuvenators help to limit permanent deformation under high-temperature loading, improving resistance to rutting and deformation in heavily trafficked pavements.

Delayed Oxidative Aging: Rejuvenating agents replenish the maltene fractions in aged binders and reduce asphaltene dominance, slowing the hardening process over the service life. This preserves the binder’s flexibility and prolongs the structural performance of the pavement.

Improved Binder–Aggregate Interaction: Rejuvenators enhance the adhesion between binder and aggregates, resulting in better coating performance and improved durability. This strengthens the mixture’s resistance to thermal, moisture, and mechanical stresses, contributing to extended pavement life.

## 4. Objectives

The study was conducted to understand asphalt’s behavior under environmental factors and to explore the effects of aging on its performance characteristics. The impact of the rejuvenator used in the binder was also examined. The mechanical properties of the original PG64-22 binder and the modified PG70-22 binder obtained from different sources were examined, and the effects of using a rejuvenator as an additive on the aging of asphalt and of using SBS polymer as a modifier on the performance of HMA were investigated. Not only the effect of using a rejuvenator as an additive on the aging of asphalt, but also the effect of using SBS polymer as a modifier on the performance of HMA was investigated. Furthermore, the trial method of applying the asphalt coating was evaluated over different periods. The main objectives of this research were to study the aging of binders and the performance of the asphalt mix. Different environmental conditions were considered. In the present study, the effects of aging on the mechanical properties of asphalt have been investigated. In the study, the impact of aging on bitumen was examined by comparing the engineering properties of bituminous hot blends before and after application. Additionally, the effect of the rejuvenator, used as an additive material, on the aging performance of bitumen was evaluated. The aged bitumen is intended to be rejuvenated and used with an additive material. In addition, the physical properties of the bitumen, which provide the binding properties of the mixture, were examined. Asphalt pavement behavior was also examined after aging. Additionally, asphalt pavement layers were analyzed with a finite element model for different environmental conditions.

In this study, the following process was used.

- Investigating binder aging progression;
- Comparison of the original and modified binder, which is used with the mix and road;
- Rheological properties of unaged and aged binders in the case of using the rejuvenator;
- The stress–strain relationship was analyzed between different layers of asphalt pavement.

The study aims to evaluate aged binders using rejuvenated additives to enable reuse in line with sustainability. In addition, the use of SBS additives in hot bituminous mixtures is expected to increase the fatigue resistance of road pavement layers under traffic and climatic conditions.

## 5. Materials and Methods

This study was designed to evaluate the performance and rheological behavior of unmodified and polymer-modified binders from different sources under short- and long-term aging, and to assess the effect of rejuvenators on aged bitumen. Three sources of PG64-22 binders and one PG70-22 binder, widely used in Illinois, USA, were selected. All binders were formulated to meet their respective PG specifications, allowing for the assessment of source-dependent aging effects while ensuring comparable performance grades. Aggregates used for asphalt mixtures included crushed stone, natural sand and recycled mixed aggregate, with gradation effects considered independently from particle shape and texture. SBS-modified bitumen was procured from TUPRAS Refinery (Kocaeli, Turkey). The aggregates incorporated in the asphalt mixtures were sourced from the Cebeci quarry (Istanbul, Turkey).

The study was conducted in four stages. In the first stage, the rheological properties and baseline performance of the original binders were determined. Rolling Thin-Film Oven Test (RTFOT) and Pressure Aging Vessel (PAV) tests were used to simulate short- and long-term aging under environmental conditions. Binder performance was assessed using dynamic shear rheometer (DSR), linear amplitude sweep (LAS), multiple stress creep recovery (MSCR), bending beam rheometer (BBR), and rotational viscosity (RV) tests. DSR and LAS tests followed ASTM D7175-23 [64] and AASHTO TP 101-14 [65] standards, respectively, using two specimens and two replicates to measure complex modulus, phase angle, and fatigue damage tolerance. MSCR tests AASHTO T350 [66] and BBR tests AASHTO T313 [67] were conducted to determine permanent deformation resistance, low-temperature stiffness, and m-value. RV tests ASTM D4402 [68] were used to assess high-temperature workability and compaction properties. Flexural creep stiffness and compliance of binders were also evaluated.

In the second stage, aged binders were treated with the rejuvenator Hydroline H90T (R1) supplied from the USA at 3%, 6%, and 9% by weight of binder. The rejuvenator type was selected based on its proven effectiveness in restoring aged binder rheological properties, while the dosage levels were chosen to represent practical application ranges and to evaluate performance sensitivity. Rejuvenator was mixed with binders aged in PAV for 5, 10, 15, 20, and 40 h, corresponding approximately to 1–20 years of field aging according to ASTM D6521 [69] and AASHTO R28 [70]. In addition, this study utilized a 60 h aging condition to simulate a precise field aging timeline and provide a comparative framework for analyzing rheological properties and rejuvenating efficacy under different aging intensities. Mixing was performed at 160 °C and 1200 rpm for 15 min to ensure homogeneity. The behavior of binders at low and high temperatures over time was assessed to evaluate rejuvenation effectiveness.

In the final stage, SBS polymer-modified and unmodified asphalt pavements were modeled using finite element analysis to evaluate stress–strain behavior of different layers under varying traffic volumes and environmental conditions, including climate. This integrated experimental and numerical approach allowed for a comprehensive assessment of binder performance, rejuvenator efficiency, and mechanistic pavement response across different aging scenarios.

In the study, the tests were applied before and after aging of the bitumen; the modified bitumen is presented below.

- Original binder, modified binder, age unmodified binder, and modified binder with 1-PAV after RTFOT to make it equivalent to aged binder;
- The aged binder is rejuvenated with Hydroline H90T (R1);
- Original binders, polymer-modified binder, and aged binders are applied to the binder performance test (DSR, BBR, RV);
- Asphalt pavement is evaluated over 1 year, and the finite element method is used to evaluate the stress–strain behavior;
- Numerical analysis of asphalt pavement.

Overall, the experimental program and numerical modeling framework were designed to provide a comprehensive evaluation of binder performance under different aging conditions and rejuvenator dosages. This integrated approach enabled a systematic assessment of rheological behavior and the structural response of asphalt pavements.

**Figure 2:**
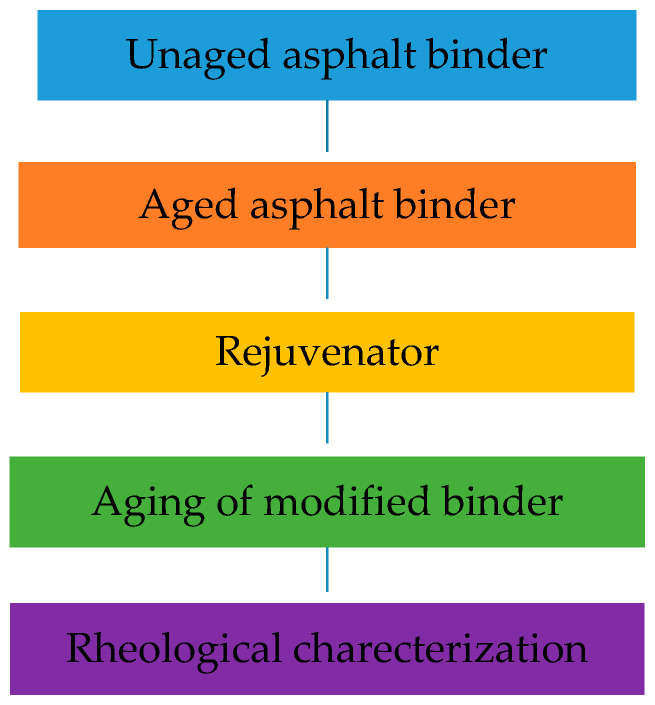
Modified binder test section.

**Figure 3:**
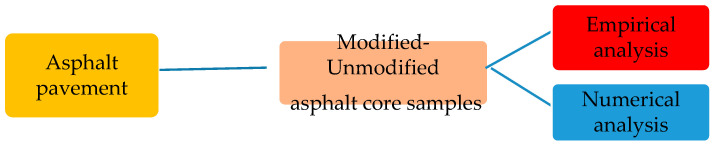
Asphalt pavement test section.

The plans for the three different sources of unmodified bitumen and one modified bitumen that were carried out in two stages are presented in Figure 2. In addition, the unmodified and modified asphalt pavement test section is given in Figure 3.

## 6. Results

The results indicate that aging significantly increased the stiffness and reduced the flexibility of the binders. The addition of the rejuvenator Hydroline H90T reduced binder stiffness and partially restored the rheological properties of aged binders. Increasing rejuvenator dosage improved flexibility and fatigue resistance, particularly in severely aged binders. Furthermore, the finite element analysis demonstrated that the changes in binder properties with SBS influenced the stress–strain response of asphalt pavement layers under traffic loading.

### 6.1. PG Grade of Different Sources of Binder

The DSR test was performed to determine the binding PG gradients obtained from different sources (AASHTO R 49-09) [71].

**Figure 4:**
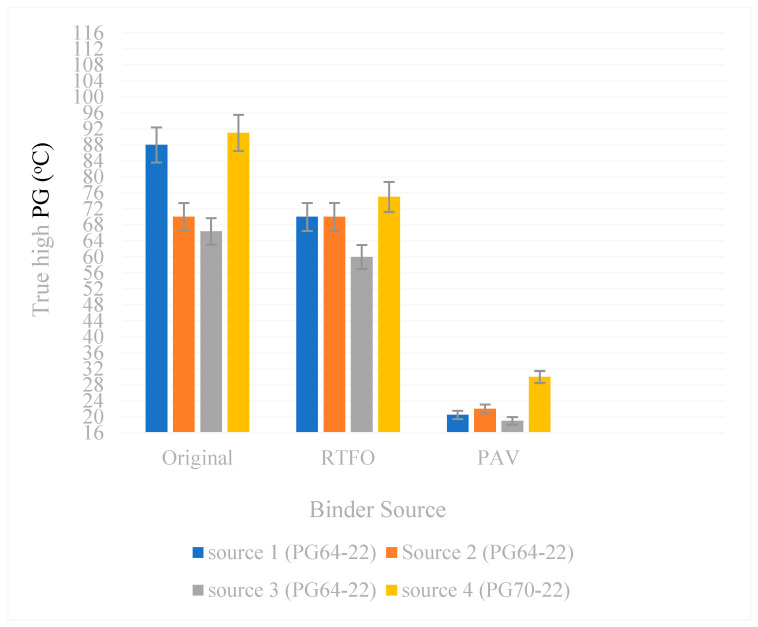
True high PG of Source 1, Source 2, Source 3, and Source 4 binders.

The true high-temperature performance grade of the original short-term-aged (rolling thin-film oven aged to simulate oxidation during HMA production) and long-term-aged (pressure aging vessel) binders from different sources is shown in Table 4 and compared in Figure 4. The actual high-temperature performance grades for the PG64-22 yielded an official high PG grade of 64 °C for source 1, source 2 and source 3 binders. The actual high-temperature performance grades for the polymer-modified binder PG70-22 yield are also seen in the high PG grade for Source 4.

The data in Table 4 illustrate the high-temperature performance characteristics of binders from four different sources, including the original binder, short-term aging via RTFOT, long-term aging via PAV, and the resulting true and standard PG grades. The original binder temperatures varied substantially among sources, from 66.4 °C for Source 3 to 91.0 °C for Source 4, indicating inherent differences in binder composition and initial stiffness. Following short-term aging, high-temperature resistance changed differently across sources, with Source 3 showing the least variation and Source 4 experiencing a pronounced increase, reflecting its greater susceptibility to oxidative hardening. Long-term aging further accentuated these differences, particularly for Source 4, which reached a PAV value of 30 °C, highlighting more significant stiffness development over the service life. Despite these differences, the final true PG grades align closely with standard classifications: PG64 for Sources 1–3 and PG70 for Source 4. These findings demonstrate that both the initial binder properties and their aging behavior strongly influence high-temperature performance, underscoring the importance of considering source-specific characteristics when designing asphalt mixtures to ensure optimal deformation resistance and long-term durability.

### 6.2. Asphalt Mass Loss

The asphalt was supplied in the same penetration grade, with modified asphalt from different sources. A short-term aging process (RTFOT) was performed, and the mass loss of asphalt was measured according to the AASHTO T240 standard [72].

**Figure 5:**
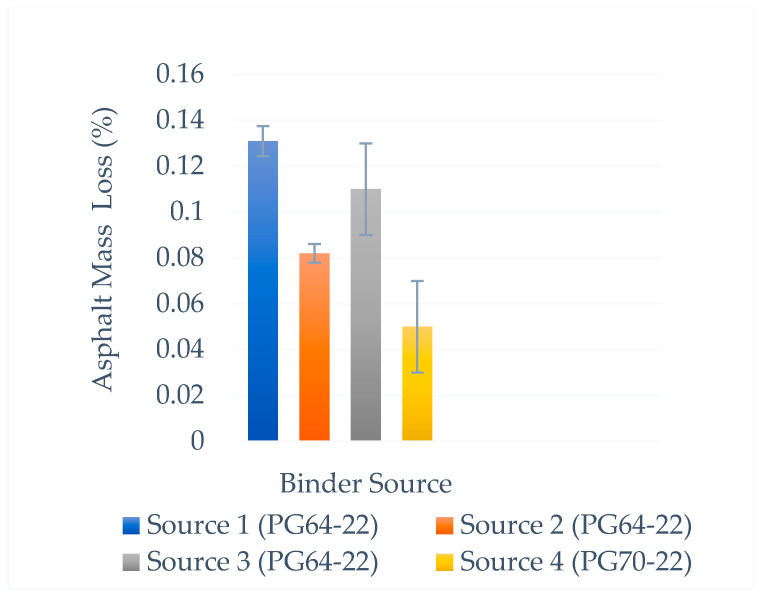
Asphalt mass loss for different binder sources.

The test results are shown in Figure 5. The test results are included in the specification limits. The asphalt mass loss value of Source 2 is less than 37%, and Source 3 is less than 16% compared with Source 1. Source 4 has the lowest asphalt mass loss because it uses a polymer-modified binder. Test results establish specification limits.

### 6.3. Linear Amplitude Sweep Test of Binder Sources

The recently developed accelerated asphalt binder fatigue test, the linear amplitude sweep (LAS) test, was conducted by AASHTO TP101-12 [73].

The linear amplitude sweep was used to estimate the fatigue resistance of asphalt binders, as specified in AASHTO TP101-12. The amplitude sweep was conducted using the dynamic shear rheometer (DSR). Kim et al. [74] proposed the following calculation method to determine the parameters.

(1) $$D(t)\approx \sum_{i=0}^{N}{\left[\pi I_{D}\gamma_{0}^{2}(|G^{*}|sin\delta_{i-1}-|G^{*}|sin\delta_{i})\right]}^{\frac{\alpha }{1+\alpha }}{\left(t_{i}-t_{i-1}\right)}^{\frac{1}{1+\alpha }}$$

where:

$I_{D}$ = initial value $\left|G^{*}\right|$ from the 1.0% applied strain interval, MPa;

$\gamma_{0}$ = applied strain for a given data point, %;

$\left|G^{*}\right|$ = complex shear modulus, MPa;

α = alpha parameter; and

T = testing time, s.

(2) $$\left|G^{*}\right|\sin\delta =C_{o\;}-C_{1}{\left(D\right)}^{C_{2}}$$

where:

$C_{0}$ = average value of $\left|G^{*}\right|\sin\delta$ the 0.1% strain interval; and

C_1_ and C_2_ = curve-fit coefficients.

According to Hintz et al. [75], the formula is shown below.

(3) $$\log\left(C_{0}-\left|G^{*}\right|\sin\delta \right)=\log(C_{1})+C_{2}\log(D)$$

The value of D_(t)_ at failure; D_f_ is defined as the D_(t)_ that corresponds to a 35% reduction in undamaged $\left|G^{*}\right|\sin\delta \left(C_{0}\right)$.

The damage parameter at failure D_f_ is defined as the value of D_(t)_ corresponding to a 35% reduction relative to the undamaged $\left|G^{*}\right|\sin\delta \left(C_{0}\right)$.

(4) $$D_{f}=\left(0.35\right){\left(\frac{C_{0}}{C_{1}}\right)}^{\left(\frac{1}{C_{2}}\right)}$$

The following binder fatigue performance model parameters are calculated as follows;

(5) $$A_{35}=\frac{f{\left(D_{f}\right)}^{k}}{k{\left(\pi I_{D}C_{1}C_{2}\right)}^{\alpha }}$$

(6) $$N_{f}=A_{35}{({Ɣ}_{max})}^{-B}$$

where:

*f =* loading frequency (10 Hz)’

k = 1 + (1 − C_2_) ɑ;

*B* = 2ɑ; and

γ_max_ = maximum expected binder strain.

To determine the parameter ɑ, the following calculations are performed from frequency sweep test data. Each frequency is converted to storage modules.

(7) $$G^{\prime }(\omega )=\left|G^{*}\right|(\omega ).\cos\delta (\omega )$$

(8) $$\log G^{\prime }(\omega )=m(\log\omega )+b$$

(9) $$\alpha =1+\frac{1}{m}$$

The linear amplitude sweep (LAS) test was performed under strain control, with strain amplitudes set from 0.1% to 10% for both the original and long-term aged binders. The test was conducted across low- and high-temperature ranges and various frequencies, as summarized in Table 5, to capture the binder’s response under conditions representative of field traffic and climatic variations. From the LAS tests, the damage parameter (Df) and fatigue life (Nf) were calculated using the viscoelastic continuum damage (VECD) approach, which quantifies the progression of micro-structural damage and estimates the number of cycles to fatigue failure. These parameters provide practical insight into the binder’s fatigue resistance and its contribution to the long-term performance of asphalt pavements. By linking rheological measurements to mechanistic performance, the LAS results offer a clearer understanding of how aging and rejuvenation influence binder durability under repeated loading.

**Figure 6:**
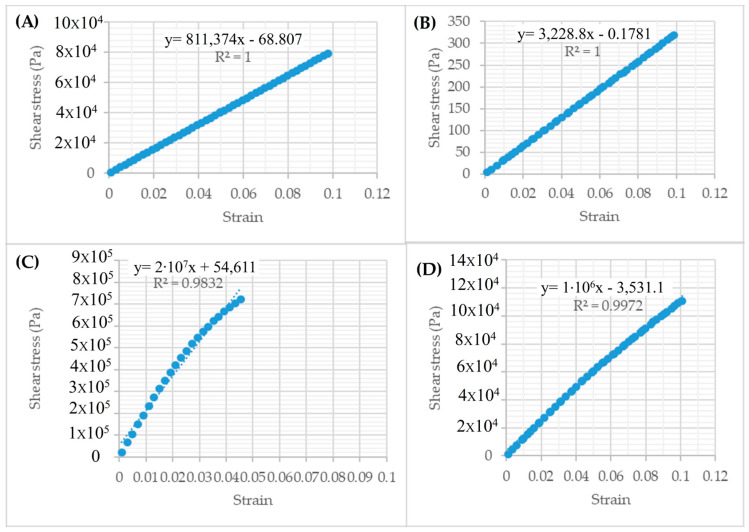
Original PG64-22; IT-HF (**A**), IT-LF (**B**), LT-HF (**C**), LT-LF (**D**) shear stress–strain linearity.

**Figure 7:**
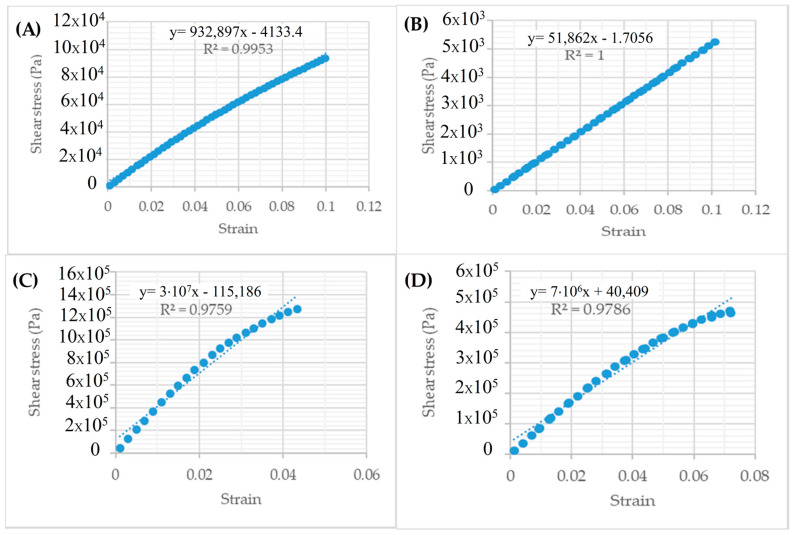
PG64-22 after PAV 1; IT-HF (**A**), IT-LF (**B**), LT-HF (**C**), LT-LF (**D**) shear stress–strain linearity.

The test results are shown in Figure 6 and Figure 7.

In the strain–stress relationship, the LAS test parameter is presented in Table 6.

Regarding the LAS test results, the binder properties changed due to long-term aging. The binder hardened due to aging, and the maximum stress increased. Due to hardening, the brittle binder showed a slight increase in maximum deformation at higher stress levels.

LAS test results show that strain values remained within the 1% tolerance during the strain-controlled frequency sweep test.

### 6.4. Fatigue Resistance of Asphalt Binders Using the LAS

The amplitude sweep test was performed in accordance with AASHTO T315 [76]. Frequency and amplitude sweep tests were performed to determine the rheological properties of the binder and the material-damage characteristics, respectively.

**Figure 8:**
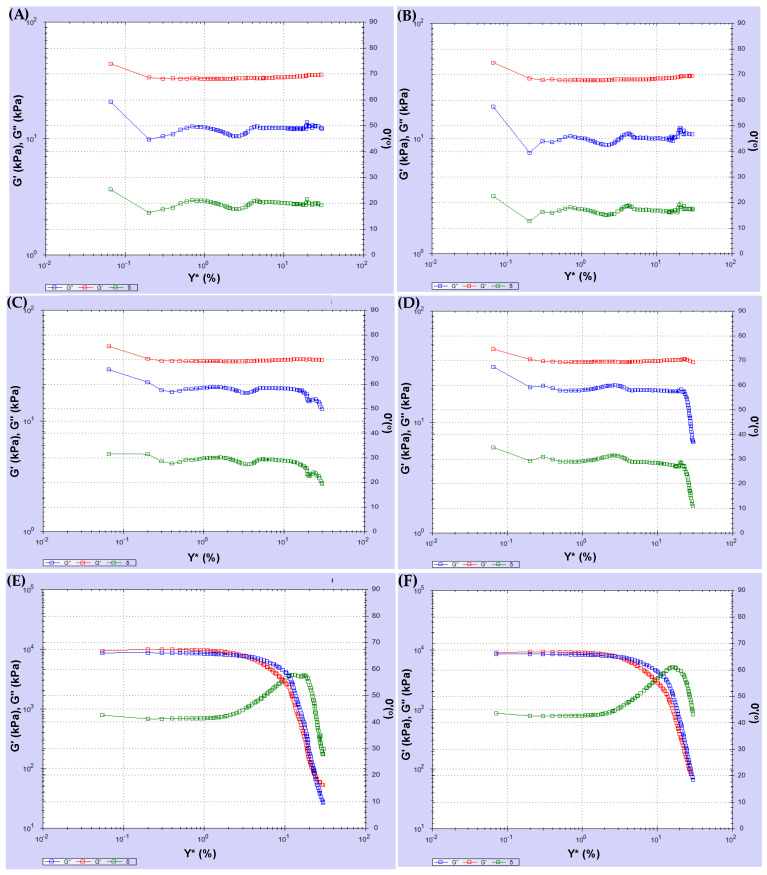
Source 1–Source 2 original binders (**A**,**B**), after RTFOT (**C**,**D**), after 1 PAV (**E**,**F**), elastic modulus, viscous modulus, and phase angle vs. shear strain treatment.

**Figure 9:**
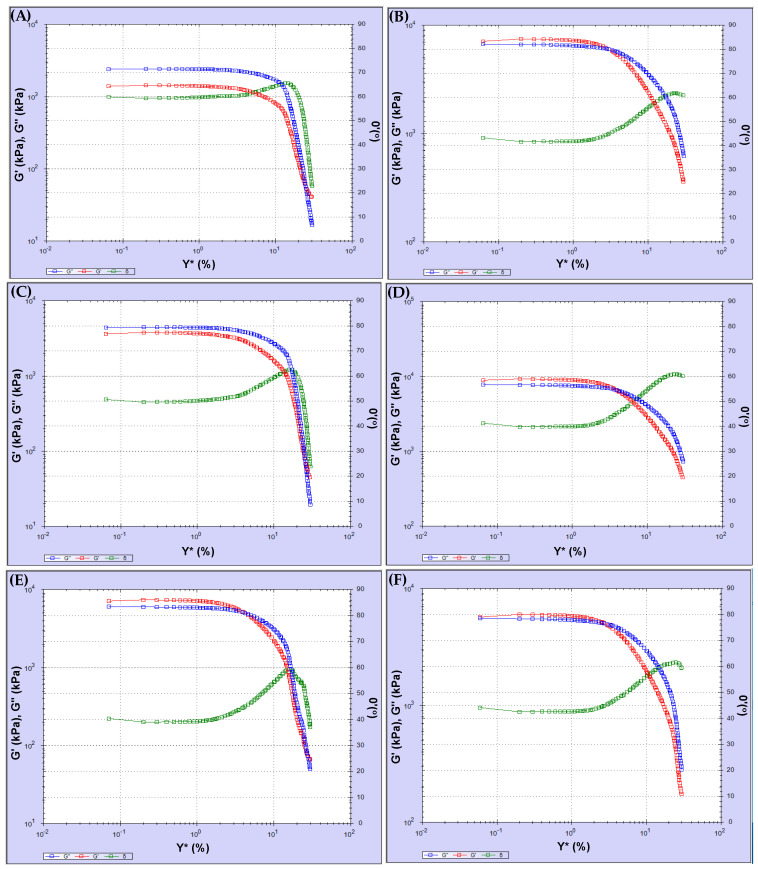
Source 3–Source 4 original binders (**A**,**B**), after RTFOT (**C**,**D**), after 1 PAV (**E**,**F**), elastic modulus, viscous modulus, and phase angle vs. shear strain treatment.

The amplitude sweep test was conducted at the selected temperature using oscillatory shear under strain-control conditions. The test was performed at 25 °C and 1% strain over a frequency range of 0.1 to 100 Hz at each temperature. The test findings revealed that complex shear strain and complex shear stress are time-dependent. Figure 8 and Figure 9 show the variations in complex shear strain and stress time.

According to Figure 8, the test results for Sources 1 and 2 were similar. When the fatigue behavior of the binder was examined across different sources, some parameters, such as the complex modulus, elastic-viscous modulus, and phase angle, decreased with increasing complex shear strain after aging. In this case, the asphalt lost its fatigue strength with aging. Referring to Figure 9, among the others, the test result for Source 3 showed greater decreases after aging. Furthermore, the phase angle of Source 4 increased after aging. It is understood that fatigue loss in the modified asphalt used in Source 4 was lower than in other sources.

### 6.5. Rheological Properties of Different Binders with Frequency Sweep Test

Source 1, source 2 and source 3 same PG grade binder, and Source 4, modified binder, were compared using the frequency sweep test. A frequency sweep test was performed under strain control. The test temperature was set between 15 °C and 45 °C, and the frequency was varied from 0.16 to 16 Hz. For the virgin binder, short- and long-term aging binder rheological properties were examined in terms of the complex modulus-frequency and phase angle-frequency correlation. The test temperature and frequency are shown in Table 7.

**Figure 10:**
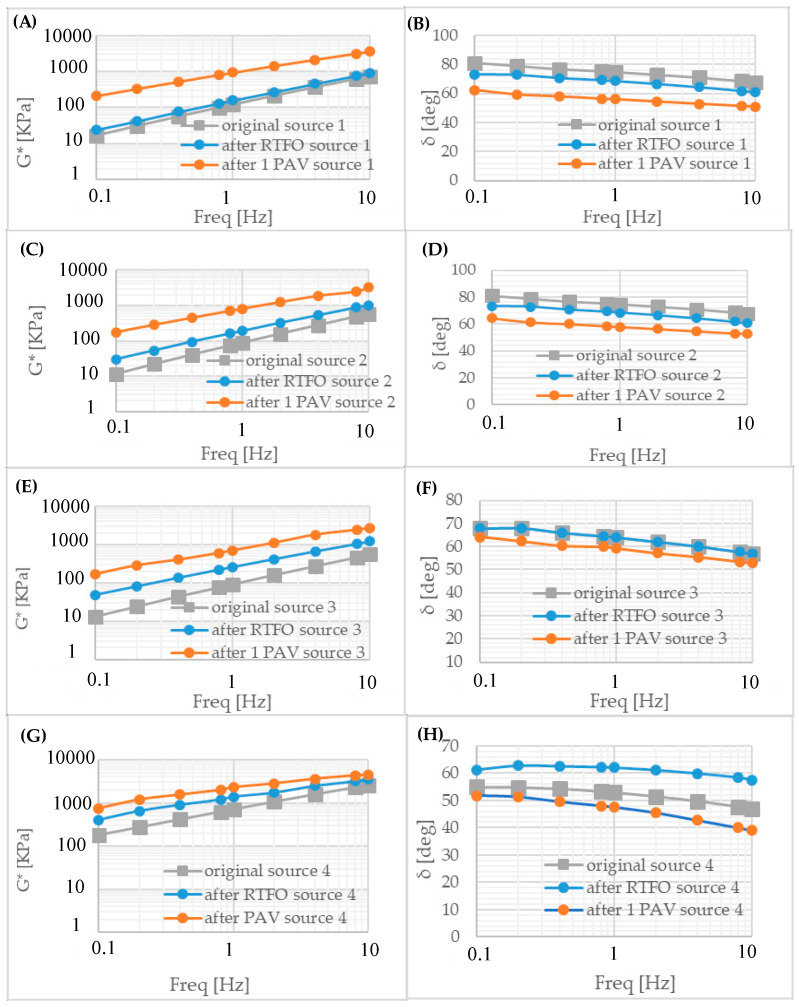
Frequency sweep test for Source 1 (**A**,**B**), Source 2 (**C**,**D**), Source 3 (**E**,**F**), and Source 4 (**G**,**H**).

The complex modulus G* and phase angle δ as a function of frequency are shown in Figure 10. At low and intermediate asphalt temperatures, the complex modulus increased, and the phase angle decreased linearly with increasing frequency. G* reached its highest value at low-temperature degrees at 15 °C, whereas δ values reached their lowest value at 15 °C. Due to the binders’ aging, G* values increased, while phase angle values decreased. Asphalt reached the highest δ value at a medium temperature (45 °C) and the lowest G* value at an intermediate temperature. Compared to Sources 1 and 2, the frequency test results were similar.

The frequency sweep test results reflect the rheological properties of bitumen before and after aging. In the frequency sweep test, the original binder and RTFOT binder values were found to be close to each other. G* values were increased, but δ values were decreased after long-term aging. Test results showed similar characteristics for Sources 1–3. The complex modulus of Source 4 was higher, and its phase angles were lower than those of the other sources. At low and intermediate temperatures, asphalt showed that complex module values increased and phase angle values decreased linearly with increasing frequency. G* reached its highest value at low-temperature degrees at 15 °C, whereas δ values reached their lowest value at 15 °C. Due to the binders’ aging, G* values increased, while phase angle values decreased. Asphalt reached the highest δ value at a medium temperature (45 °C) and the lowest G* value at an intermediate temperature. Compared with Sources 1 and 2, the frequency test results were similar.

### 6.6. Multiple Stress Creep Recovery Test for Different Sources of Binder

According to AASHTO T 350, the MSCR test is used to evaluate the rutting performance of binders [66]. The nonrecoverable creep compliance, Jnr, is calculated as a measure of the binder’s contribution to the mixture’s permanent deformation behavior.

In this test method, the elastic response of the asphalt binder under stress is determined as follows.

Calculate average percent recovery at 0.1 kPa:

(10) $$R_{0.1}=\frac{SUM\left[\varepsilon_{r}(0.1N)\right]}{10}\;\mathrm{for}\;N=11\;\mathrm{to}\;20$$

$\varepsilon_{r}$: Strain value

Calculate average percent recovery at 3.2 kPa:

(11) $$R_{0.1}=\frac{SUM\left[\varepsilon_{r}(3.2N)\right]}{10}\;\mathrm{for}\;N=1\;\mathrm{to}\;10$$

Nonrecoverable creep compliance at 0.1 kPa $J_{nr_{0.1}}$, kPa^−1^:

(12) $$J_{nr_{0.1}}=\frac{SUM\left[J_{nr}(0.1N)\right]}{10}\;\mathrm{for}\;N=11\;\mathrm{to}\;20$$

Nonrecoverable creep compliance at 0.1 kPa $J_{nr_{3.2}}$, kPa^−1^:

(13) $$J_{nr_{3.2}}=\frac{SUM\left[J_{nr}(3.2N)\right]}{10}\;\mathrm{for}\;N=1\;\mathrm{to}\;10$$

Nonrecoverable creep compliance between 0.1 kPa and 3.2 kPa is given by the following formula:

(14) $$J_{nrdiff}=\frac{\left[J_{nr3.2}-J_{nr0.1}\right]}{J_{nr0.1}}$$

**Figure 11:**
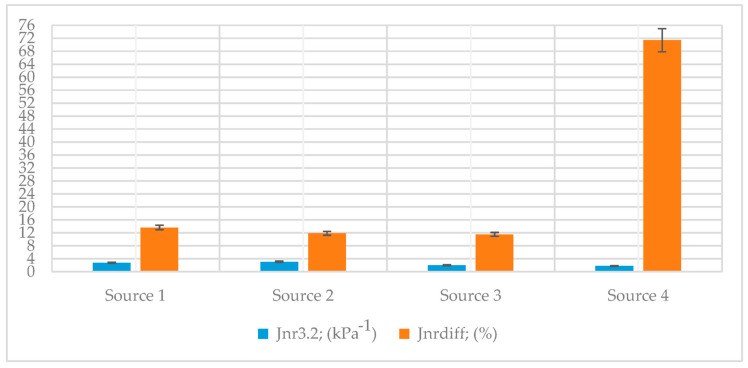
Determined different sources of PG64 22-PG70-22 nonrecoverable creep compliance.

According to AASHTO T350, the MSCR test results are shown in Table 8, and the nonrecoverable creep compliance data for Jnr3.2 and Jnrdiff are demonstrated in Figure 11.

Figure 11 shows that the creep compliance test results were similar across all binder sources (Source 1, Source 2, and Source 3). The Jnr3.2 values for Source 2 and Source 3 were approximately 12% higher than those for Source 1, indicating a slightly greater susceptibility to permanent deformation. In contrast, the Jnrdiff value for Source 1 was nearly 15% higher than that for Source 2, reflecting greater non-recoverable strain variability in Source 1. Moreover, the Jnrdiff value for Source 4 was higher than that of the other sources, which can be attributed to the use of a modified binder. These results suggest subtle but notable differences in the elastic and viscoelastic responses among the binder sources.

### 6.7. Flexural Creep Stiffness and Compliance of Bituminous Materials with BBR Test

The bending beam rheometer (BBR) test method covers the determination of the flexural creep stiffness and compliance of bituminous materials using a BBR (AASHTO T 313) [67]. The tests were performed at the Illinois Center for Transportation laboratory in Rantoul, Illinois, USA. It was conducted to evaluate the rheological properties of binders at low temperatures in accordance with Superpave specifications.

Using the elementary bending theory, the deflection of an elastic beam can be obtained by applying the following equation:

(15) $$\delta =\frac{PL^{3}}{48EI}$$

δ: deflection of the beam at midspan, mm;

P: load applied, N;

L: span length, mm.

E: modulus of elasticity, MPa; and

I: the moment of inertia, mm^4^.

The linear viscoelastic stiffness modulus is assumed to follow the following relationship with the stiffness:

(16) $$S(t)=\frac{PL^{3}}{4bh^{3}\delta (t)}$$

S(t): time-dependent flexural creep stiffness, MPa;

P: constant load, N;

L: span length, mm;

b: width of the beam, mm;

h: the thickness of the beam, mm;

δ(t): the deflection of the beam, mm.

The BBR test was performed on different sources of the same PG grade (PG64-22) and PG70-22 binders. The original PG 64-22 and the modified PG70-22 binder were used after the long-term aging (1 PAV) procedure. The test temperature was −12 °C according to AASHTO M 320 [77].

The test results for stiffness and m-value at 60.0 s are given in Table 9.

**Figure 12:**
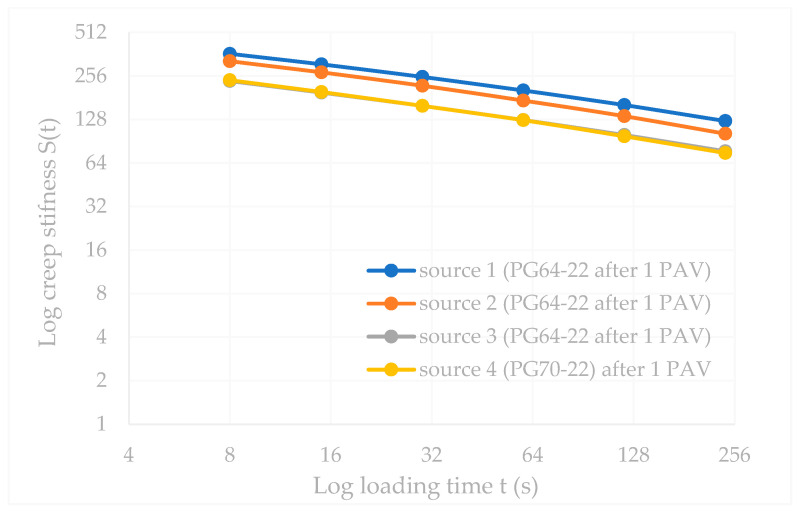
Assessment of creep stiffness with varying loading times.

It is noted that the creep stiffness depends on the loading time, as shown in Figure 12. The stiffness values exhibit a time-dependent parallelism for Sources 1, 2, 3, and 4, respectively.

**Figure 13:**
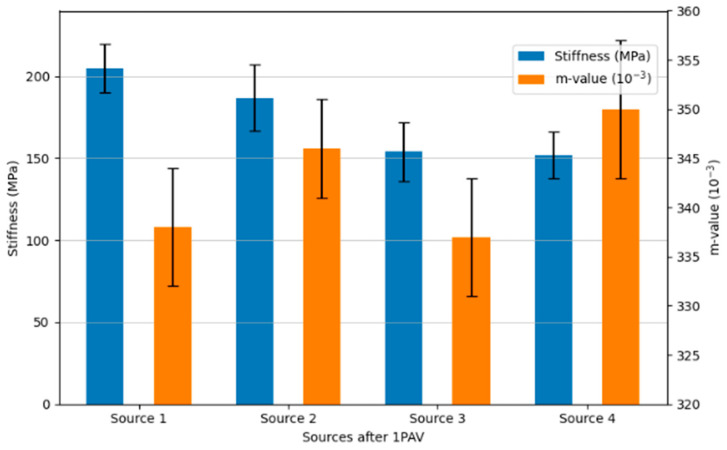
Stiffness and m-values of different sources of the same PG64-22 long-term aging binder.

The stiffness and m-value ratios for the sources are shown in Figure 13.

In compliance with Figure 13, the stiffness value of Source 1 is 17.3% higher than Source 2 and 59.84% higher than Source 3, respectively, whereas the m-value ratio of Source 2 is 7.69% higher than both Source 1 and Source 3. Despite having the same PG rating, there are minor differences among the three sources. However, Source 4 has the highest m-value among the same PG binder sources.

### 6.8. The Viscosity of Different Sources of Binder

The RV test was applied to determine the viscosity of asphalt binders during production and application. As regards AASHTO T 316 [78] and ASTM D 4402 [68], a rotational viscometer was used to determine the viscosity of the asphalt binder.

The RV test provides information on the viscosity of the asphalt binder during pumping and mixing [68,78].

The cP value obtained in the formulation is converted to Pa·s:

cP × 0.001 = Pa⋅s(17)

Viscosity expressed in terms of angular velocity is determined using the following equation.

η = τ /γ(18)

τ = T/(2πRs^2^ L) (19)

γ = [2 ω Rc^2^ Rs^2^)]/[x^2^ (Rc^2^ − Rs^2^)] (20)

where:

η = dynamic viscosity (Pa·s);

τ = shear stress (N/cm^2^);

γ = shear rate (s^−1^);

T = torque (in N·m);

L = effective spindle length (m);

Rs = spindle radius (m);

Rc = container radius (m);

ω = rotational speed (radians/s);

x = radial location where shear rate is being calculated (m).

A viscosity test was performed on virgin, short-term-aged, and long-term-aged binders from different sources. The test was conducted at 105 °C, 135 °C, and 165 °C, respectively. Rotational viscometer test results are shown in Table 10 and Table 11.

**Figure 14:**
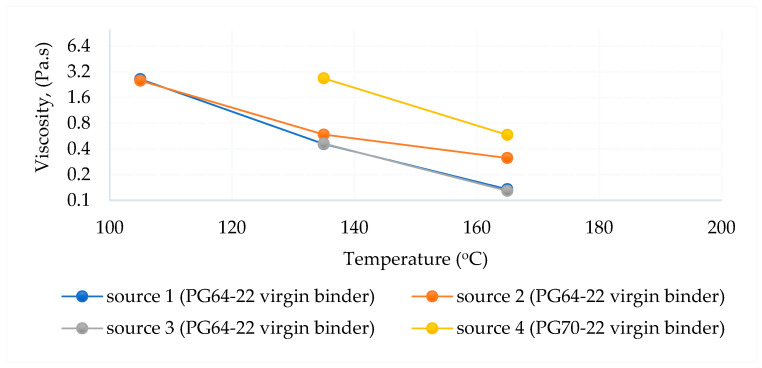
Typical viscosity curve for virgin binder.

**Figure 15:**
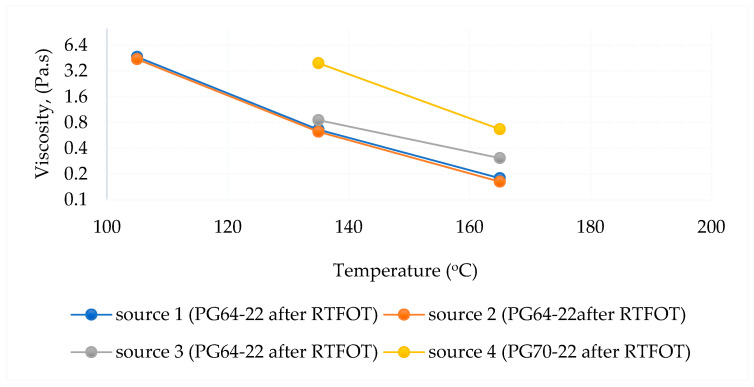
Typical viscosity curve for short-term aging.

**Figure 16:**
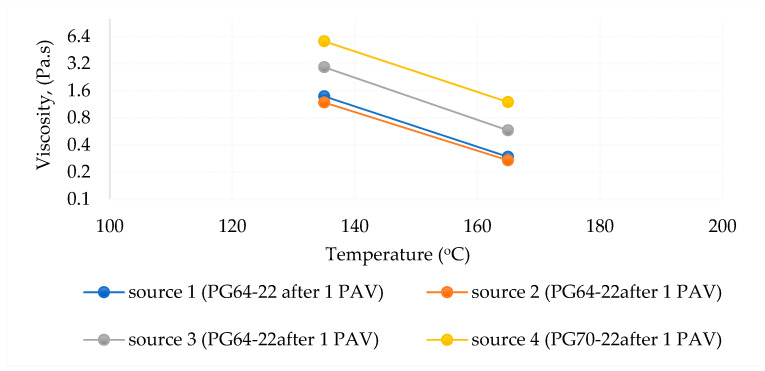
Typical viscosity curve for long-term aging.

The viscosity–temperature relationships are shown in Figure 14, Figure 15 and Figure 16, respectively. According to the test results, the viscosity–temperature relationships of all binders were similar before and after aging. However, Source 4 has the highest viscosity among the same PG binder sources.

**Figure 17:**
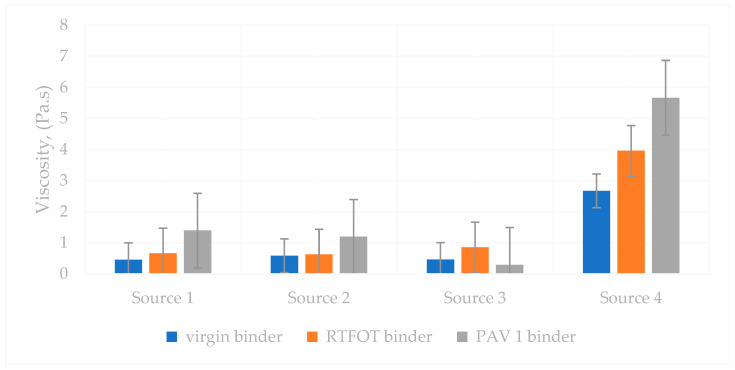
The viscosity of different aging types of binder (PG64-22 and PG70-22).

The different aging status of the PG64-22 and PG70-22 binders is shown in Figure 17.

As shown in Figure 17, the viscosities of asphalt from different sources (Sources 1, 2, and 3 for the PG64-22 binder) were initially similar prior to aging; however, significant variations emerged following both short-term and long-term aging. These differences were particularly pronounced at elevated testing temperatures, reflecting the influence of source-specific binder composition on oxidative hardening and rheological response. The results indicate that while the original binders exhibit comparable flow behavior, aging processes amplify source-dependent disparities in stiffness and deformation resistance, which can directly affect the performance and durability of asphalt pavements under service conditions. Source 4 has higher viscosity due to the use of PG70-22 binder. It is understood that it increased in viscosity with age and complied with the AASHTO standard specification. In addition, the viscosity of Source 4 is higher than the standard after 1 PAV.

### 6.9. Properties and Specifications

The control binders were tested in accordance with AASHTO T315 [76]. According to this standard, the pre-aging and short-term post-aging properties of binders at high temperatures are determined. In long-term aging, the low-temperature properties are determined by AASHTO T313 [67].

The results are shown in Table 12. After short and long-term aging, PG70-22 (Source 4) viscosity values exceeded the standard limit. PG 64-22 and PG70-22, used in experimental studies on results other than these, meet the AASHTO M320 standard [77].

As shown in Table 12, when considering the physical properties of PG64-22 bitumens with the same penetration degree obtained from different sources before aging, a difference of 22.55% occurred between the lowest (Source 1) and highest (Source 2) viscosity values. This difference increased by 26.82% between the lowest (Source 2) and highest (Source 4) values in short-term aging and by 79.08% between the lowest (Source 3) and highest (Source 1) values in long-term aging.

When comparing PG64-22 with the same penetration degree (Sources 1, 2, 3) with PG70-22 with different penetration degrees (Source 4), the largest difference before aging was 82.82%; in short-term aging it was 84.22%, and in long-term aging it was 95%.

Considering the low-temperature performance characteristics, even at the same penetration degree, a difference of 37.44% is observed between the lowest (Source 3) and highest (Source 1) stiffness values of PG64-22 bitumens obtained from different sources, and similarly, a difference of 4.69% is observed between the lowest (Source 1) and highest (Source 2) m-value values.

When comparing Source 1 and Source 4, an increase in penetration degree results in a 37.93% decrease in stiffness and a 7.41% decrease in m-value at low temperatures. Therefore, even with the same penetration degree, empirical analyses of bitumens obtained from different sources reveal differences in their low-temperature performance characteristics. An increase in penetration degree, when considering low-temperature performance analyses, leads to a decrease in stiffness values.

### 6.10. Assessment of Rheological Properties of Binder Using the Rejuvenator

**Figure 18:**
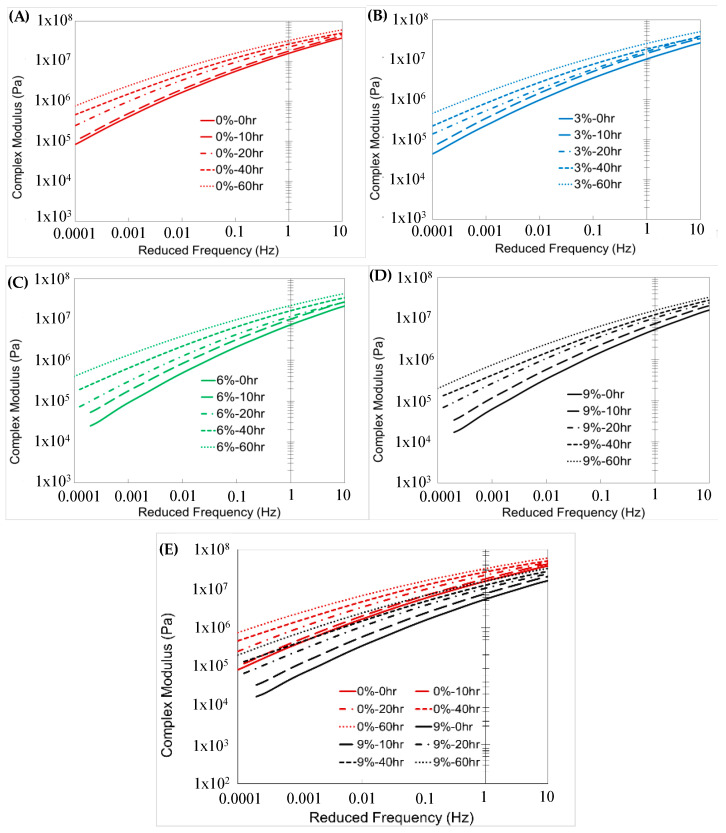
Complex modulus master curves for different aging conditions and rejuvenator contents (**A**) 0%, (**B**) 3%, (**C**) 6%, (**D**) 9%, and (**E**) comparison of 0% to 9%.

A frequency sweep test with DSR was performed on aged binders. The test was conducted at low and medium temperatures (from 15 °C to 45 °C). The binders were initially aged to 1-PAV to simulate field aging, and subsequently subjected to PAV aging for 0, 10, 20, 40, and 60 hours, with rejuvenator dosages of 0, 3, 6, and 9% (Figure 18).

The master curves were constructed using the Christensen–Anderson model and shifted to a 15 °C reference temperature [80]. Two replicates were used to determine the final master curve of each material.

The effect of aging is evident from the binders’ complex modulus master curves in Figure 18. For non-rejuvenated samples, the master curve of phase angles shifts downward due to both aging and the stiffer binder. Non-rejuvenated binders age and harden over time as the main curve shifts upward. On the other hand, the main curve downward, and binder hardening decreases with rejuvenation due to aging.

From a mechanistic standpoint, the rejuvenator restores the maltene fraction reduced by oxidative aging, thereby improving molecular mobility and reducing binder stiffness, as reflected by the downward shift in the complex modulus master curves.

### 6.11. Glover-Rowe (G-R), Crossover Frequency, and Rutting Parameter

**Figure 19:**
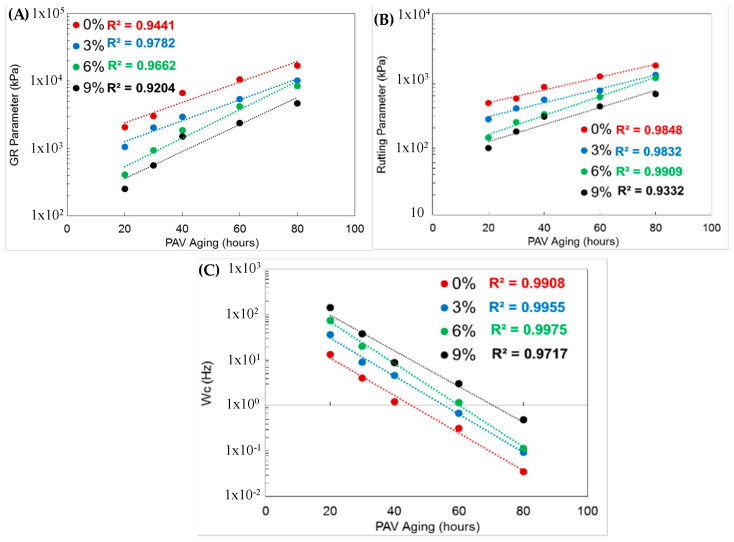
Variation of (**A**) G-R parameter, (**B**) dutting parameter, and (**C**) crossover frequency with aging.

G-R, crossover frequency, and rutting parameters were derived from the DSR test results. The calculated parameters for different aging conditions and rejuvenation applications are presented in Figure 19. The results show that the G-R and rutting parameters increased. At the same time, the frequency decreased with the aging of the binders. There is a strong correlation across all sample test results, with correlation coefficients exceeding 0.90. The binder becomes brittle with age. The results show an R^2^ of 0.92 and a strong correlation between the parameters. Samples with different rejuvenator contents also followed a similar trend. In addition, rejuvenation softens the binder by altering its chemical structure. Figure 19B shows the effects of rejuvenator use on aging behavior. The use of rejuvenation has positive effects on G-R and rutting parameters. Even under extreme aging conditions, the use of rejuvenators yielded better performance than non-use. There is an opposite trend at the crossover frequency.

Based on the rheological characterization results, the addition of a rejuvenator makes the binder softer and shows its effect even after prolonged laboratory aging (10, 20, 40 and 60 h of PAV). Also, with increasing rejuvenation content, a reduction in binder stiffness is observed.

### 6.12. Numeric Analysis

The finite element method was applied in numerical analysis studies. In the finite element method, the road superstructure is divided into elements using least-squares, and the stiffness matrix of each element is computed; the solution is then obtained by integrating all the elements. The mechanical properties of the stress and deformation behaviors of the whole structure are obtained using differential equations, and the equations are solved numerically in matrix form. In the finite element method, the behavior of complex structures is determined using computer programs [81,82].

Plaxis is among the most widely used programs for FEM. The Plaxis 2D Version 8 program was preferred in the study to examine the behavior of soil layers under load.

**Figure 20:**
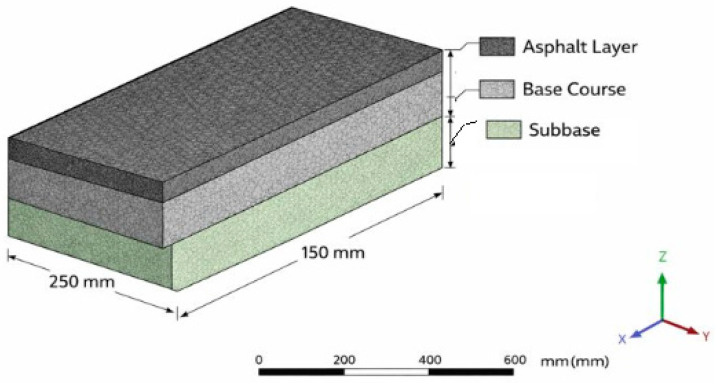
Finite element model (FEM) of the road, adapted from [83], Elsevier, 2017.

The FEM model of the road is shown in Figure 20.

### 6.13. Plaxis Finite Element Model and Loading Process with Boundary Conditions

**Figure 21:**
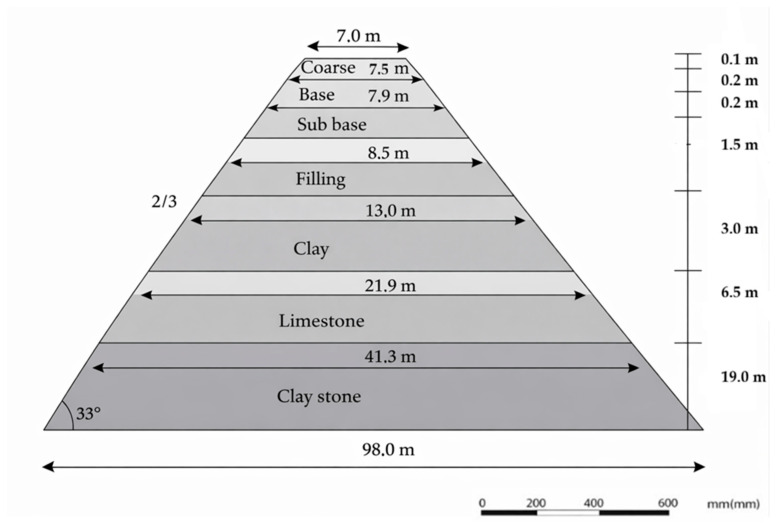
Road filling section.

The superstructure and natural ground layers of the road consist of seven layers, as shown in the road filling section of Figure 21. The natural ground layer comprises the filling, clay, limestone, and claystone formations.

**Figure 22:**
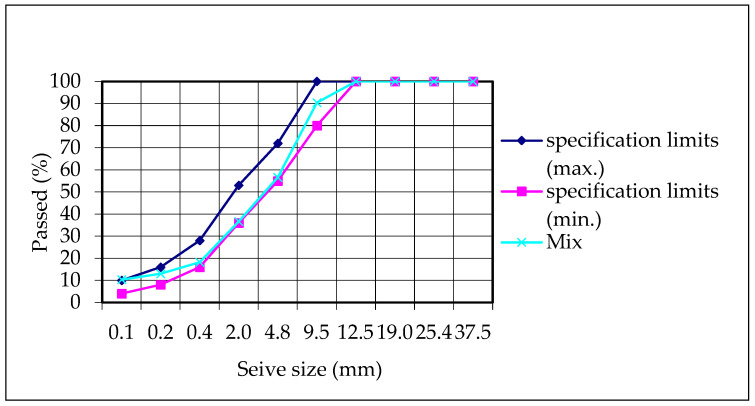
Granulometry of SBS-modified HMA mixture.

The modified bitumen and aggregate properties and the aggregate gradation used in the HMA mixture are presented in Table 13 and Table 14 and Figure 22, respectively. The aggregate and modified bitumen used in the mix met the specification limits.

The SBS-modified HMA design properties are given in Table 15.

Numerical analysis was performed using FEM, with parameters obtained from experimental studies of the superstructure and ground layer under traffic and environmental conditions over one year. To form a solid model, the Plaxis Finite Elements Program was used. The actual situation and the solid model were generated using parameters from drilling core samples. These laboratory findings are consistent with the FEM predictions, which indicate reduced tensile stresses and delayed damage accumulation in SBS-modified asphalt pavements, independent of rejuvenator effects, under traffic and environmental loading.

The design parameters of the superstructure road are presented in Table 16.

**Figure 23:**
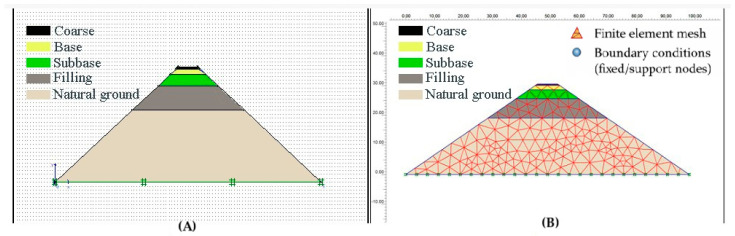
Model geometry (**A**), finite element mesh, and boundary condition (**B**).

The model geometry and finite element mesh are shown in Figure 23.

**Figure 24:**
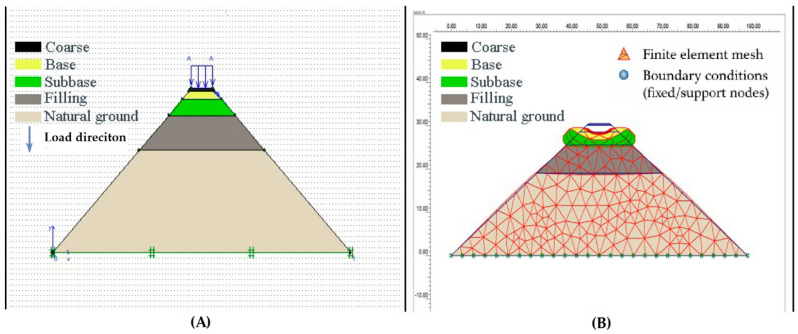
Geometric model on loading condition (**A**); geometric model deformed on finite element mesh (**B**).

After the finite element network and boundary conditions were established, traffic loading was analyzed. The loading condition designated and the finite element mesh model deformed with charging are shown in Figure 24. 

**Figure 25:**
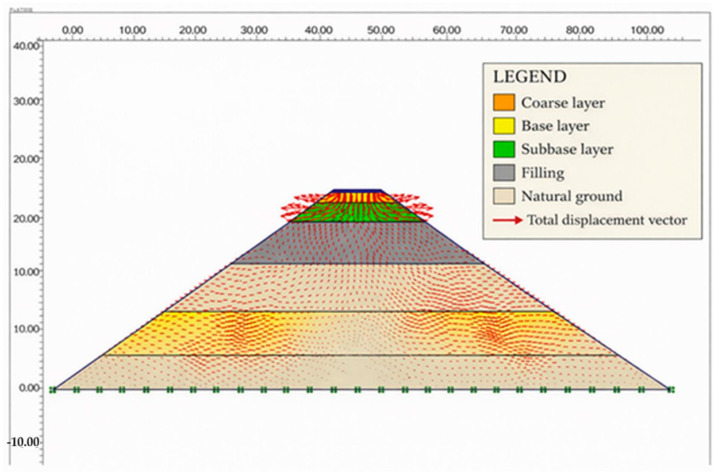
Total displacement vector.

**Figure 26:**
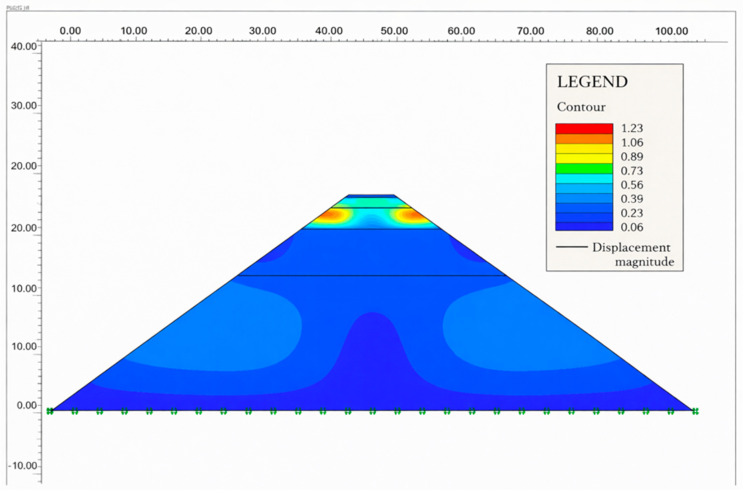
Total displacement contour.

The superstructure road and subsoil layer formed by the fill are shown as the maximum displacements in the sections where the fill and clay layers are located in Figure 25 and Figure 26. 

**Figure 27:**
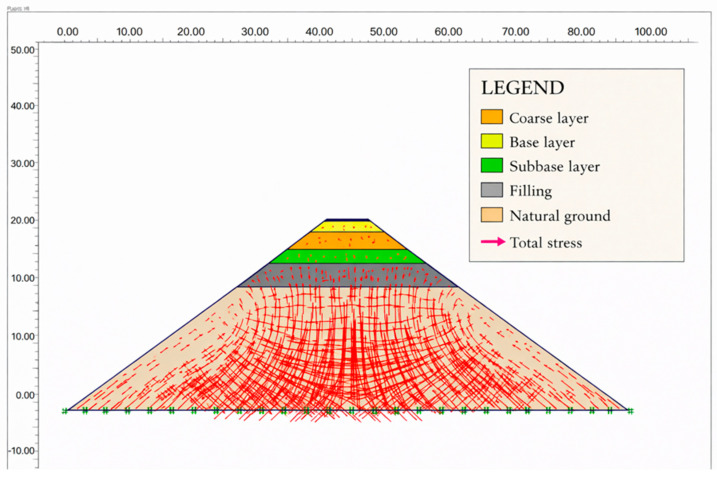
Total stress on the superstructure road and subsoil layer.

**Figure 28:**
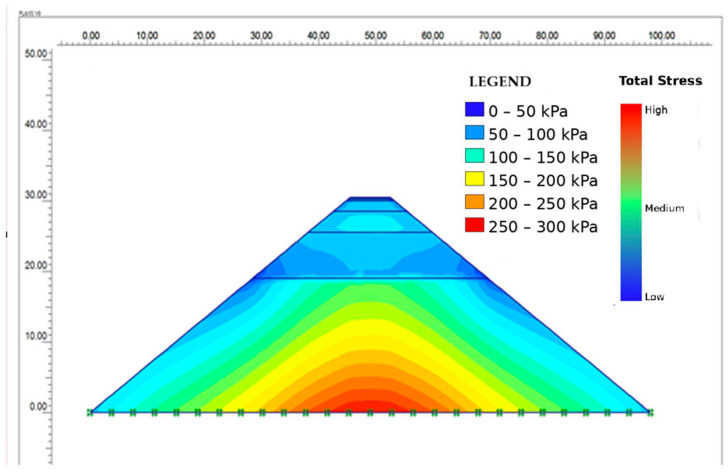
Total stress contour.

The superstructure road and subsoil layer, formed by the filling and stress vectors, are shown in Figure 27 and Figure 28. Stresses in the filler layers have spread to the base.

### 6.14. Total Stress and Displacement Changes with Plaxis

Monthly changes in total stress and displacements in unloaded and traffic-loaded sections were considered on the road superstructure and subsoil layers.

**Figure 29:**
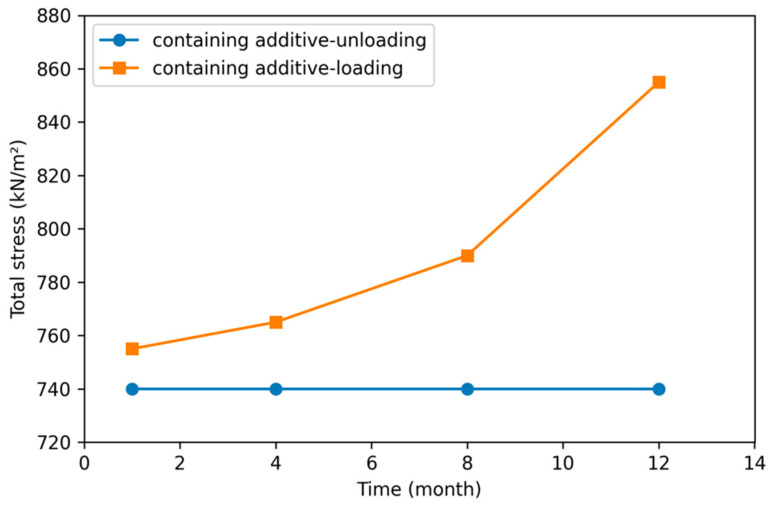
Total stress-time changing on pavement and soil containing additives with loading and unloading situations.

The monthly variation in the total stresses in the additive equivalent axle-loaded and uncharged superstructure road and the subsoil layers is shown in Figure 29.

According to Figure 29, the total stress on the road superstructure and subsoil layers with the SBS additive increased linearly with increasing traffic loading. There is no change in unloading traffic conditions over the past year.

**Figure 30:**
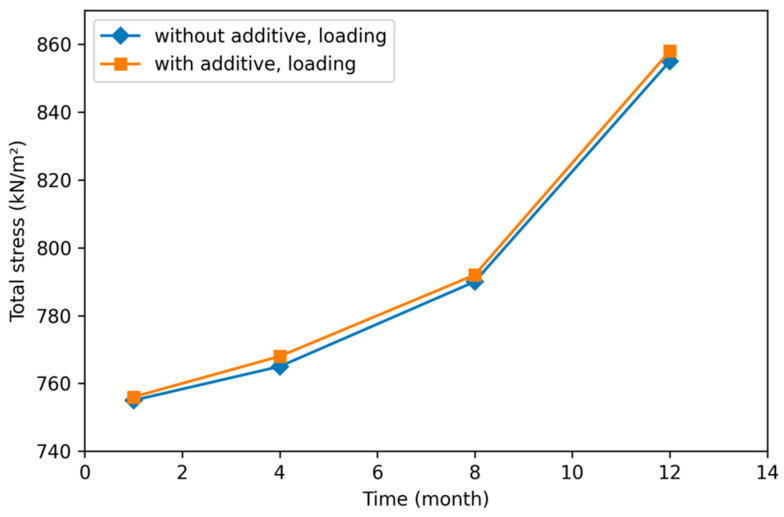
Total stress–time changing under loading on pavement and soil containing additives and without additives.

Asphalt pavement, with equivalent standard axle loads with and without an additive, and the monthly total stress changes in the road sublayers were compared in Figure 30.

When Figure 30 is examined, it is seen that the changes in the total stress values of the superstructure of the road and the subsoil layers exposed to equivalent axle load are similar and increase linearly with monthly changes.

The total displacement changes in the road superstructure and sublayers are given in Table 17.

**Figure 31:**
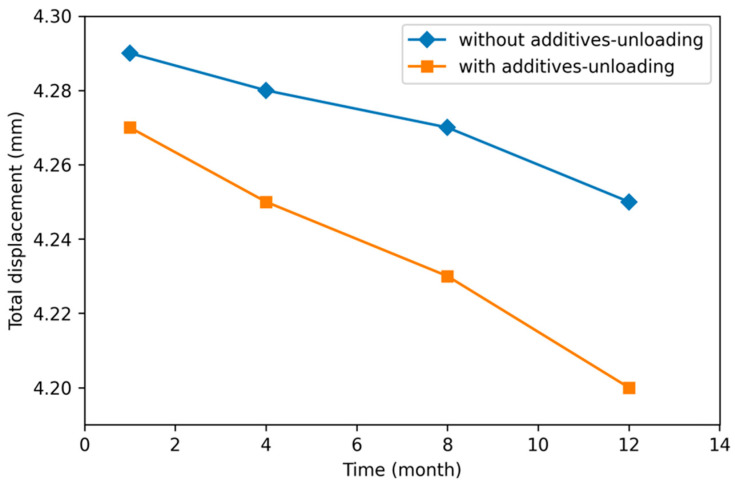
Total displacement changes with the unloading condition.

**Figure 32:**
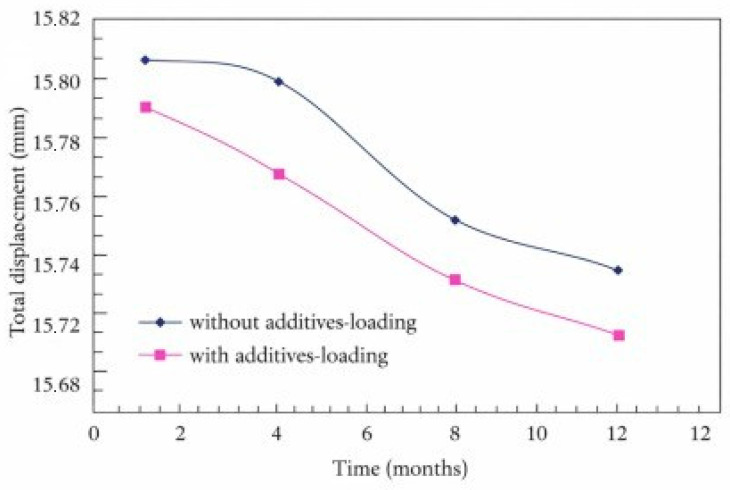
Total displacement changes with the loading condition.

The total displacement of unmodified and modified asphalt pavements with SBS and sublayers, under equivalent axle loading and without loading conditions, is shown in Figure 31 and Figure 32, respectively.

In Figure 31, because the edge part of the road is not exposed to wheel loading, the total change in the linearity of the road layer and sublayer soil due to self-weight is seen to decrease.

In the annual period, the variation in total displacement decreased to 0.10 mm and 0.4 mm, respectively, in the soil layers without additives and with additives under unloading conditions. It was observed that total displacement was greater with additives during soil layer unloading.

When Figure 32 is examined, it is seen that the total displacement changes without additives-loading and with additives-loading asphalt pavement and sublayers exposed to their specific weights, and equivalent vehicle axle load decreases linearly by the same amount, 0.09–0.10 mm annually.

## 7. Discussion

The findings of this study demonstrate that asphalt aging and rejuvenation behavior cannot be adequately described using generalized assumptions. Despite sharing the same PG classification, the binders exhibited markedly different aging sensitivities depending on their source, with differences becoming more pronounced under long-term aging conditions. This observation extends previous studies that typically assume equivalent aging behavior for binders within the same grade.

Rejuvenator effectiveness was found to be strongly dependent on aging severity. While significant stiffness reduction was observed at early aging stages, the ability of the rejuvenator to restore rheological properties diminished as aging progressed, resulting in partial rather than full recovery for heavily aged binders. This aging-level-dependent response highlights the limitations of applying uniform rejuvenation strategies without considering aging history.

The rheological trends observed at the binder level were consistently reflected in the mechanistic pavement analyses, where aging-induced stiffening increased pavement stiffness and stress concentrations, while rejuvenation led to more favorable stress redistribution. These results emphasize the importance of integrating binder source variability, aging level, and rejuvenation effects in performance-based pavement evaluation.

Although all binders investigated in this study share the same PG classification, the rheological results clearly demonstrate that aging sensitivity varies significantly depending on binder source. In particular, the rate of increase in complex modulus and viscosity with progressive aging differed among the binders, indicating that chemical composition and refinery origin play a critical role in aging evolution.

Previous studies have generally assumed comparable aging behavior for binders within the same performance grade and often relied on a single binder source for evaluation [1,93]. More recent studies have begun to acknowledge source effects; however, these are typically limited to a single aging condition [94,95]. The present study extends the existing literature by demonstrating that source dependency becomes more pronounced under long-term aging, suggesting that binder selection based solely on PG grading may overlook critical durability differences.

The effectiveness of the rejuvenator was found to be strongly dependent on the aging level of the binder. While rejuvenator addition significantly reduced stiffness and viscosity at early and intermediate aging stages, its ability to restore rheological properties diminished as aging severity increased. In heavily aged binders, rejuvenation led to partial recovery rather than full restoration to original binder properties.

Many previous studies have reported the softening effect of rejuvenators and their potential to improve workability and cracking resistance [96,97]. However, these studies typically evaluate rejuvenation at a single aging condition or assume uniform effectiveness across aging levels. The findings of this study demonstrate that rejuvenator efficiency is not constant, but instead exhibits diminishing returns with increasing aging severity, likely due to irreversible oxidative and molecular changes in the binder structure. This highlights the importance of considering aging history when selecting rejuvenator dosage and application strategy.

The rheological trends observed at the binder level were reflected in the mechanistic pavement analyses. Changes in binder stiffness due to aging and rejuvenation translated into measurable variations in layer modulus, stress distribution, and deformation response under traffic and environmental loading. Stiffer aged binders resulted in increased pavement stiffness and higher stress concentrations, whereas rejuvenated binders showed reduced modulus and more favorable stress redistribution.

While mechanistic–empirical models are widely used to link binder properties to mixture and pavement response, previous studies often consider aging, polymer modification, or rejuvenation independently [98]. The present study integrates these factors and demonstrates that source-dependent aging and aging-level-dependent rejuvenation can significantly influence predicted pavement performance. This integrated approach strengthens the practical relevance of the rheological findings and supports their use in performance-based pavement design.

The rheological trends observed at the binder level due to SBS polymer modification were consistently reflected in mechanical pavement analyses, where polymer-induced stiffening increased pavement stiffness and stress concentrations. These results underscore the importance of considering SBS modification and binder source variability in performance-based pavement assessment.

The numerical analysis results demonstrate that SBS modification yields mechanically meaningful improvements in asphalt pavement response under combined traffic and climatic loading. Pavement sections incorporating SBS-modified asphalt exhibited lower stress concentrations and more uniform stress distribution within the structural layers, particularly at elevated service temperatures. The viscoelastic contribution of the SBS polymer enhanced resistance to deformation under repeated traffic loading, as reflected in reduced critical strain levels within the pavement structure. Although aging altered the mechanical properties of the asphalt layers, the SBS-modified pavement consistently exhibited a more favorable mechanical response compared to the unmodified configuration, indicating the effectiveness of SBS modification in enhancing pavement performance.

## 8. Conclusions and Recommendations

In this study, binders with identical PG grades obtained from different sources were evaluated to investigate source-dependent rheological and aging behavior. The results showed that unaged and short-term aged binders from different sources exhibited comparable rheological properties. However, under long-term aging conditions, binders with the same PG grade demonstrated markedly different aging sensitivities depending on their source. Rejuvenator effectiveness was found to decrease with increasing aging severity, leading to only partial recovery in heavily aged binders. These rheological changes have a direct influence on the mechanistic response of asphalt pavements under traffic loading, emphasizing the importance of accounting for aging history in performance evaluation. Numerical analyses further confirmed that SBS polymer modification significantly enhances pavement mechanical performance under combined traffic and climatic conditions. The inclusion of SBS-modified asphalt resulted in reduced stress and strain demands and improved resistance to deformation within the pavement structure. Overall, the findings highlight the necessity of jointly considering binder source, aging level, rejuvenation strategy, and polymer modification when assessing the long-term performance of asphalt pavements.

The results indicate that binders from different sources exhibit comparable rheological behavior in the unaged and short-term aged conditions. However, under long-term aging, distinct performance differences become evident, primarily driven by source-dependent chemical evolution associated with oxidation and hardening processes. While the present study captures these differences at the rheological level, a more detailed chemical characterization—particularly through SARA fraction analysis (saturates, aromatics, resins, and asphaltenes)—is essential to fully explain the observed divergence in long-term aging behavior. Such analyses would enable a clearer linkage between compositional changes and performance degradation, and provide a stronger mechanistic basis for evaluating binder durability across different sources.

Furthermore, the Hydroline H90T (later expressed as R1) ensured that the additive was investigated as a potential rejuvenator. The binder samples were prepared at different rejuvenator dosages and aged at various PAV rates to study the effect of aging on the binder with and without rejuvenation. The effect of adding 3%, 6%, and 9% rejuvenator by weight of binder on the rheological properties of the PG 64-22 sourced was studied. The following results were obtained in the study.

- The rejuvenator had a positive effect on the improvement of the asphalt’s low-temperature properties.
- Rejuvenator use reduced aging.
- At low rejuvenator contents, G* slightly increases due to aging conditions, whereas at higher rejuvenator contents (9%), G* decreases compared to the non-rejuvenated binder for the same aging level. Rejuvenation increases and decreases the G* parameter for specific aging conditions.
- Aging increased with time.
- The gap between 0 hr and 60 hr increases with increasing 9% rejuvenator.
- The G-R parameter increases with aging.
- The use of SBS improves the durability of asphalt under traffic and environmental conditions.

The results reveal two key observations. First, although rejuvenated binders initially exhibit reduced stiffness, progressive aging leads to a gradual increase in stiffness, indicating a partial reduction in rejuvenation effectiveness over time. While this reduction is not pronounced, it highlights the aging sensitivity of rejuvenated systems. Second, these findings suggest that evaluating rejuvenation performance using a single rejuvenator (e.g., R1) may not fully capture the variability in long-term behavior. A broader assessment incorporating different types of rejuvenators with varying chemical compositions is therefore necessary to more comprehensively understand their aging resistance and long-term performance effectiveness. Furthermore, the mechanical response of pavement structures is governed by the interaction of multiple layers with distinct material characteristics, whose behavior under loading varies significantly with traffic intensity and environmental conditions, particularly climate-related effects over time. In addition, the properties of the underlying ground layers play a critical role in overall pavement performance. Therefore, to achieve a comprehensive understanding of stress–deformation relationships and long-term performance across pavement layers, numerical analyses should be systematically integrated with empirical and laboratory-based investigations. Such combined approaches provide a more robust framework for performance-based pavement design and maintenance decision-making.

In future studies, performance tests should be conducted on both loose mixtures and extracted core samples at multiple time intervals within the test sections. Additionally, bitumen and binder blend performance tests should be carried out to evaluate the long-term behavior of the core samples under varying environmental conditions. Flexibility index tests are recommended for different test sections at 24, 36, 48, and 60 months. Moreover, bitumen extraction tests should be performed on the core samples to assess aging effects, and chemical analyses should be used to determine the evolving chemical properties of the bitumen.

The test steps are illustrated below.

- Core samples are used to implement extraction tests to determine aging properties at different time points.
- Core samples are collected using the I FIT test (top-down) for environmental conditions at different times.
- Bitumen is characterized chemically after an extraction test.
- Binders are applied to the binder performance test (direct tension test).
- The behavior of asphalt in traffic and environmental conditions will be examined by using different additives.
- The properties of asphalt coatings in heavy traffic and harsh climatic conditions will be compared under different additive treatments.

## Figures and Tables

**Table 1:** List of individual factors and conjoint factors affecting asphalt aging, reproduced from [57], University of Nottingham, 2009.

Factors	Influenced By					Occuring	
	Time	Heat	Oxygen	Sun-Light	Beta & Gamma Rays	At the Surface	In Mass
Oxidation (in dark)	+	+	+			+	
Photo-oxidation (direct light)	+	+	+	+		+	
Volatilisation	+	+				+	
Photo-oxidation (reflect light)	+	+	+	+		+	
Photo-chemical (direct light)	+	+		+		+	
Photo-chemical (reflected light)	+	+		+		+	+
Polymerization	+	+				+	+
Steric or physical	+					+	+
Exudation of oils	+	+				+	
Changes by nuclear energy	+	+			+	+	+
Action by water	+	+	+	+		+	
Absorption by solid	+	+				+	+
Absorption of components at a solid surface	+	+				+	
Chemical reactions	+	+				+	+
Microbiological deterioration	+	+	+			+	+

**Table 2:** Bitumen modification types, adapted from [62,63], ISFALT, 2001, and Thomas Telford Publishing, 2003.

Modification Types	Specimens
I. Modification with non-polymer additive a. Filler b. Anti-peel additives c. Expander (extenders) d. Antioxidants e. Organo-metal compounds f. Others	Clay, Black Carbon, Fly Ash Organik Aminler ve Amidler Lignin and Sulfur Zinc and Lead Antioxidants, Phenolics Amines Organo Manganese Compositions Organo Carbon Compositions
II. Modification with polymer additives a. Plastics 1. Thermoplastics 2. Thermosets b. Elastomers 1. Natural Rubbers 2. Artificial Elastomers 3. Processed Rubbers 4. Fibers	Polyethylene (PE), Polypropylene (PP), Polyvinyl Chloride (PVC), Polystyrene (PS), Ethylene Vinyl Acetate (EVA) Epoxy Resins Synthetic Butadiene Copolymer (SBR), Styrene Butadiene Styrene Copolymer (SBS), Ethylene Prokplendien Harmoliper (EPDM), Isobutene Isoprene Copolymer (IIR) Polyester, Fibers, Polypropylene Fibers
III. Chemical Reaction Modification	Additive Reaction (bitumen + monomer) Volcanization (bitumen + sulfur) Nitrogen Reaction (bitumen + nitric acid)

**Table 3:** Benefits of different types of modifications, reproduced from [63]. Thomas Telford Publishing, 2003.

Modifier	Permanent Deformation	Thermal Cracking	Fatigue Crack	Moisture Damage	Aging
Elastomers	+	+	+		+
Plastomers	+				
Processed Rubber		+	+		
Black Carbon	+				+
Lime					+
Sulfur	+				
Chemical Modifier	+				
Antioxidants					+
Hydrated Lime				+	+

**Table 4:** True high PG of different source types on binders.

Source Type	Original Binder (°C)	RTFOT (°C)	PAV (°C)	True Grade (°C)	PG Grade (°C)
Source 1	88.00	70	20.5	88	64
Source 2	70.00	70	22	70	64
Source 3	66.4	60	19	66	64
Source 4	91.0	75	30	91	70

**Table 5:** Original and PAV binder LAS test parameter.

Binder Type	Properties	Temperature (°C)	Frequency (Hz)
Low-temperature	Low-temperature low frequency (LT-LF)	15	0.16
	Low-temperature high frequency (LT-HF)	15	16
	High-temperature low frequency (HT-LF)	45	0.16
	High-temperature high frequency (HT-HF)	45	16

**Table 6:** LAS test max (stress–strain) parameter.

Binder Type	Properties	Stress–Strain	
		Max Stress (Pa)	Max Strain
Original PG 64-22	IT-HF (45 °C 16 Hz)	79,465	0.09796
	IT-LF (45 °C 0.16 Hz)	318.6	0.09878
	LT-HF (15 °C 16 Hz)	7.22 × 10^5^	0.04551
	LT-LF (15 °C 0.16 Hz)	110,950	0.1008
Long-term aged PG 64-22 (PAV 1)	IT-HF (45 °C 16 Hz)	93,705	0.09997
	IT-LF (45 °C 0.16 Hz)	5249	0.1016
	LT-HF (15 °C 16 Hz)	1,275,000	0.04336
	LT-LF (15 °C 0.16 Hz)	462,950	0.072125

**Table 7:** Frequency sweep test properties.

Temperature	Frequency	
(°C)	Minimum (Hz)	Maximum (Hz)
15	0.16	16
25		
35		
45		

**Table 8:** MSCR test results for different sources.

Binder Sources	Source 1	Source 2	Source 3	Source 4	AASHTO Standard
					T350
Sample description	PG 64-22 (s)1	PG 64-22 (s)2	PG 64-22 (s)3	PG 70-22 (s)4	
Nonrecoverable creep compliance at 0.1 kPa, Jnr_0.1_, kPa^−1^	2.419	2.753	1.776	1.022	
Nonrecoverable creep compliance at 3.2 kPa, Jn_r3.2_, kPa^−1^	2.749	3.079	1.981	1.752	Max 4.5 kPa^−1^
The percent difference between nonrecoverable creep compliance Jnr_diff_, (%)	13.65	11.85	11.5	71.4	Max 75%
Percent recoverable strain at 0.1 kPa, (%)	5.0	3.4	7.1	51.9	
Percent recoverable strain at 3.2 kPa, (%)	1.2	0.9	2.3	28.9	
The percent difference between nonrecoverable creep compliance (%)	76.5	72.7	67	44.3	
Average creep strain at 0.1 kPa	0.255	0.285	0.1912	0.2127	
Average creep strain at 3.2 kPa	8.90	9.94	6.489	7.89	
Average end strain at 0.1 kPa	0.2414	0.2753	0.1776	0.1022	
Average end strain at 3.2 kPa	8.798	9.852	6.338	5.607	
Average recoverable strain at 0.1 kPa	0.01271	0.00966	0.01357	0.1104	
Average recoverable strain at 3.2 kPa	0.10235	0.09192	0.1518	2.283	
Percent nonrecoverable strain at 0.1 kPa, (%)	95.0	96.6	92.9	48.1	
Percent nonrecoverable strain at 3.2 kPa, (%)	98.85	99.1	97.7	71.1	

**Table 9:** BBR test result parameters.

Source	Measured Stiffness (MPa)	m-Value	Deflection (mm)	P Force (mN)
1	225	0.316	0.36235	1009.5
1-1	192	0.325	0.42046	1003.6
1-2	191	0.334	0.42404	1005.3
2	145	0.33	0.56283	1013.5
2-1	201	0.352	0.40535	1012.2
2-2	173	0.369	0.46698	1002.1
3-1	131	0.339	0.60902	988.3
3-2	123	0.336	0.64968	988.5
4-1	129	0.349	0.61898	988.3
4-2	124	0.352	0.64246	984.7

**Table 10:** A different source of PG64-22 binder viscosity test results.

Source		1	2	3	1	2	3	1	2	3
Temperature		105 °C			135 °C			165 °C		
Binder Type	Test Measurement									
PG64-22 virgin binder	Viscosity (cP)	2614	2513	-	456	588	462.5	135	313	129.2
	Torque (%)	20.93	20.2	-	3.65	4.7	3.7	1.1	2.4	1.1
	S Str (Pa)	17.78	17.12	-	3.10	3.91	3.14	0.92	2.1	0.94
PG64-22 after RTFOT	Viscosity (cP)	4683	4412	-	662.5	625	854.2	179	162.5	308.3
	Torque (%)	37.5	35.3	-	5.3	5.0	6.8	1.4	1.3	2.5
	S Str (Pa)	31.88		-	4.51	4.25	5.74	1.22	1.10	2.10
PG64-22 after 1 PAV	Viscosity (cP)	-	-	-	1396	1196	2925	296	271	583.3
	Torque (%)	-	-	-	11.2	9.5	11.7	2.4	2.2	2.3
	S Str (Pa)	-	-	-	9.49	8.13	16.38	1.98	1.87	3.27

**Table 11:** Source 4 PG70-22 binder viscosity test result.

Source		4	4	4
Temperature		105 °C	135 °C	165 °C
Binder Type	Test Measurement			
PG70-22 binder	Viscosity (cP)	-	2675	583.3
	Torque (%)	-	21.5	4.7
	S. Str. (Pa)	-	18.24	3.96
PG70-22 after RTFOT	Viscosity (cP)	-	3962.7	670.8
	Torque (%)	-	31.6	5.4
	S. Str. (Pa)	-	26.78	4.56
PG70-22 after 1 PAV	Viscosity (cP)	-	5666.7	1200
	Torque (%)	-	22.7	4.8
	S. Str. (Pa)	-	31.78	6.72

**Table 12:** Properties of different-source unaged and aged control binders.

Asphalt Property	Units	Source 1	Source 2	Source 3	Source 4	Method	AASHTO M320
Flash point (C.O.C.)	°C	-	-	-	-	AASHTO T48 [79]	230 min
Apparent viscosity for the original binder at 135 °C	Pa·s	0.456	0.588	0.462	2.67	AASHTO T316 [78]	3.00 max
Apparent viscosity for binder after RTFOT at 135 °C	Pa·s	0.662	0.625	0.854	3.96	AASHTO T316	3.00 max
Apparent viscosity for binder after 1 PAV at 135 °C	Pa·s	1.396	1.196	0.292	5.66	AASHTO T316	3.00 max
Mass change, RTFOT residue	Mass %	0.131	0.082	0.107	0.185	AASHTO T240 [72]	1.00 max
BBR (stiffness) at −12 °C	MPa	203	173	127	126	AASHTO T313 [67]	300 max
BBR (m-value) at −12 °C	-	0.325	0.341	0.337	0.351	AASHTO T313	0.300 min

**Table 13:** SBS-modified bitumen physical properties.

Property	Value	Test Method
Spesific gravity (g/cm^3^)	1.017	EN 15326 [84]
Penetration (0.1 mm, 100 g, 5 s.)	B-68	EN 1426 [85]
Softening point (°C)	54.0	EN 1427 [86]
Ductility (25 °C, cm)	>100	EN 13589 [87]
Fraas breaking point (°C)	−17.5	EN 12593 [88]

**Table 14:** Aggregate properties for HMA.

Property	Value	Test Method
Los Angeles abrasion loss (%)	20.5	ASTM C131 [89]
Bulk specific gravity of coarse aggregate	2.733	ASTM C127 [90]
Bulk specific gravity of fine aggregate	2.678	ASTM C128 [91]
Bulk specific gravity of mineral filler	2.764	ASTM D854 [92]
Absorption coarse aggregate (%)	0.38	ASTM C127
Absorption fine aggregate (%)	0.88	ASTM C127

**Table 15:** Modified HMA design properties.

Property	SBS-Modified HMA
Optimum bitumen rate (%)	5.24
Practical specific gravity (g/cm^3^)	2.411
Marshall stability (kgf)	1170
Flow (mm)	3.58
Aggregate void ratio (%)	13.9
Asphalt void ratio (%)	75.4
Air void ratio (%)	3.10

**Table 16:** Model parameters in unmodified and SBS-modified superstructure road [83].

Material	Period (Month)	Equivalent Axle Load, (t) Unmodified/Modified	Elasticity Modulus, (MPa) Unmodified/Modified	Poisson’s Ratio Unmodified/Modified	Density, (t/m^3^) Unmodified/Modified
Course	1	451/451	2879/3105	0.35/0.35	2.267/2.271
	4	1328/1328	3221/4089	0.35/0.35	2.272/2.287
	8	2563/2563	6824/8058	0.35/0.35	2.277/2.289
	12	3091/3091	8052/9636	0.35/0.35	2.284/2.288

**Table 17:** Total displacement values on the pavement and sublayer with Plaxis finite element analysis.

Month	Unmodified-Unloading (mm)	Unmodified-Loading (mm)	Modified-Unloading (mm)	Modified-Loading (mm)
1	4.29	15.81	4.27	15.79
4	4.28	15.80	4.25	15.76
8	4.27	15.74	4.23	15.71
12	4.25	15.72	4.20	15.69

## Data Availability

The original contributions presented in this study are included in the article. Further inquiries can be directed to the corresponding author.
